# Effects of Modern Food Retailers on Adult and Child Diets and Nutrition

**DOI:** 10.3390/nu12061714

**Published:** 2020-06-08

**Authors:** Makaiko G. Khonje, Olivier Ecker, Matin Qaim

**Affiliations:** 1 Department of Agricultural Economics and Rural Development, University of Goettingen, 37073 Goettingen, Germany; mqaim@uni-goettingen.de; 2 International Food Policy Research Institute (IFPRI), 1201 I Street, NW, Washington, DC 20005, USA; o.ecker@cgiar.org

**Keywords:** child undernutrition, overweight, obesity, food environments, supermarkets, Africa

## Abstract

In many developing countries, food environments are changing rapidly, with modern retailers—such as supermarkets—gaining in importance. Previous studies have suggested that the rise of modern retailers contributes to overweight and obesity. Effects of modern retailers on dietary quality have not been analyzed previously due to the unavailability of individual-level dietary data. Here, we address this research gap with data from randomly selected households in Lusaka, Zambia. Anthropometric and food-intake data from 930 adults and 499 children were analyzed to estimate effects of purchasing food in modern retailers on body weight, height, and dietary quality while controlling for income and other confounding factors. The food expenditure share spent in modern retailers was found to be positively associated with overweight in adults, but not in children. For children, a positive association between expenditures in modern retailers and height was identified. Modern retailers contribute to higher consumption of ultra-processed foods and calories. But they also increase protein and micronutrient intakes among adults and children, mainly through higher consumption of meat and dairy. The findings underline that modern retailers can influence diets and nutrition in positive and negative ways. Differentiated regulatory policies are needed to shape food environments for healthy food choices and nutrition.

## 1. Introduction

Malnutrition is a global problem with serious negative health implications [1,2]. Especially in developing countries, different types of malnutrition typically coexist within the same communities, households, and even individuals [3,4,5,6]. While undernutrition and micronutrient deficiencies remain widespread, overweight and obesity are rapidly on the rise [1,2,3,4,5,6]. Food environments, defined as the physical, economic, and sociocultural context in which consumers acquire their food, can influence dietary choices and nutrition [5,7,8]. Food environments are changing rapidly [8,9]. In many developing countries, modern retailers—including supermarkets, hypermarkets, convenience stores, and fast-food restaurants—are gaining in importance at the expense of traditional food markets and shops [9,10]. Due to higher efficiency and economies of scale, modern retailers may improve consumers’ access to affordable foods [10,11,12]. On the other hand, modern retailers tend to sell more processed foods than traditional markets [1,8,13]. Highly processed foods are often rich in fat, sugar, and salt, but poor in micronutrients. Hence, the growth of modern retailers in developing countries may increase calorie consumption without necessarily improving dietary quality. Possibly, modern retailers may even worsen dietary quality through promoting the consumption of unhealthy snacks, beverages, and convenience foods [5,8,14].

A few recent studies have analyzed the effects of modern retailers on diets and nutrition in developing countries. Most of these studies focused on the role of supermarkets. Studies in Guatemala, Indonesia, Kenya, and Thailand showed that purchasing food in supermarkets is associated with a higher consumption of processed and ultra-processed foods [15,16,17,18]. In Guatemala and Kenya, supermarket food purchases were also shown to be associated with rising body mass index (BMI) and increased prevalence rates of overweight, obesity, and related chronic diseases among adults [15,17,19,20].

Effects of modern retailers on child nutrition were rarely analyzed. One recent study with data from Kenya showed that supermarkets do not contribute to childhood obesity but have a positive effect on child linear growth and height [21]. Positive effects on child height are surprising, as linear growth is known to be closely related to dietary quality and balanced nutrient intakes [3,22]. Do supermarkets and other modern retailers contribute to improved nutrient intakes? This is an important question, which none of the previous studies were able to answer. Previous studies collected food consumption data at the household level, suggesting that purchasing food in supermarkets can lead to higher dietary diversity in some situations [21,23,24]. But household-level data neglect intra-household food distribution and are therefore not necessarily good proxies of individual-level dietary quality and micronutrient intakes.

We provide the first study that analyzed effects of modern retailers on diets and nutrition with individual-level food-intake and anthropometric data from adults and children in a developing country. We used cross-section observational data collected in urban Zambia. Like many other countries in sub-Saharan Africa, Zambia is characterized by a high prevalence of different forms of malnutrition and a rapid modernization of food environments [13,25].

Our data were collected in Lusaka, the capital city of Zambia. The number of large shopping malls in Lusaka increased from 1 in 1995 to 25 in 2018 (Supplementary Table S1). Shopping malls often include hypermarkets, supermarkets, and fast-food restaurants. In addition, the number of supermarkets and convenience stores outside of large shopping malls also grew substantially in Lusaka in recent years.

## 2. Materials and Methods

### 2.1. Survey of Households and Individuals

Data for this study were collected through a survey of households and individuals in Lusaka, Zambia. The survey was implemented between April and July 2018. We surveyed a total of 475 households using a two-stage sampling procedure. Lusaka has a total of 40 key compounds (sometimes also called sections). At the first sampling stage, out of the total of 40 compounds, we purposively selected 14, using statistics on mean income levels. We selected compounds to represent high, middle, and low mean income levels (Supplementary Figure S1). At the second sampling stage, we randomly sampled about 35 households in each of the selected 14 compounds based on household lists provided by the compound leadership. The exact number of households sampled per compound was adjusted based on total compound population numbers. Sampled households were informed about the study and were asked for their cooperation. If they agreed, an appointment was scheduled for personal interviews. Almost all of the households sampled (98%) agreed to participate; the few households that refused were replaced by other randomly selected households in the same compound.

In each sampled household, we interviewed the household head or the adult responsible for food purchase decisions and food preparation. Many of the main household respondents were female (see below). We recruited local enumerators to conduct face-to-face interviews in local languages. The enumerators were trained and supervised by the researchers. The structured questionnaire covered sections on the household demographic structure, economic activities, income, and consumption expenditures. Data on food consumption at the household level were collected through a 7-day recall using a list of 140 different food items and capturing quantities, prices, processing levels, and sources of each item.

Food-intake and nutrition data were captured at the individual level for up to four randomly selected members of each household: two adults (≥18 years) and two children/adolescents (6 months–18 years). In this article, we use the term “children” for all individuals <18 years. Individual-level food-intake data were collected through 24 h dietary recalls; for small children, the recall questions were answered by the caregiver. We carried out one 24 h recall per study participant. Doing a second recall a few days later with the same persons is useful to better capture dietary variation, but was not possible in our study due to logistical constraints.

In addition to the dietary data, we measured the weight and height of all participating adults and children. The anthropometric measures were taken following recommended practices [6]. Weight was measured without heavy clothing and shoes using a normal weighing scale. For small children, the weight of the caregiver with and without carrying the child was taken and the difference was calculated. Height was taken using a stadiometer (height board). For precision, we took each weight and height measure twice.

Overall, we collected complete individual-level dietary and anthropometric data from 930 adults (623 female and 307 male adults) and 499 children (271 girls and 228 boys) (Supplementary Table S2). All participating adults provided written informed consent for themselves and their children. The study was reviewed and approved by the Ethics Committee of the University of Goettingen on 28 February 2018 (project code RTG 1666-B3).

### 2.2. Measuring Nutrition and Dietary Quality

Several previous studies with data from other developing countries showed that the use of modern supermarkets is associated with an increase in the consumption of processed and highly processed foods [14,15,17,19]. To evaluate whether this is also true in urban Zambia, we classified all food items purchased and consumed by sample households according to their level of processing. We differentiated between unprocessed foods, primary processed foods, and ultra-processed foods [13,19]. Unprocessed foods include wholegrain cereals and pulses, fresh fruits and vegetables, eggs, and fresh milk, among others. Primary processed foods include milled cereals and fresh meat and fish. Ultra-processed foods include bread, pasta, dairy products, sausages and meat products, soft drinks, sweets, and other ready-made dishes and snacks (Supplementary Table S3). For all three processing categories, we calculated household-level food expenditure shares as indicators of dietary composition.

We also constructed several other diet and nutrition indicators using the individual-level data from adults and children. Individual nutritional status was evaluated with body height and weight measures. For adults, we calculated the body mass index (BMI), whereby BMI > 25.0 kg/m^2^ was defined as overweight or obese. For children, we calculated BMI-for-age *Z*-scores (BAZ) and height-for-age *Z*-scores (HAZ) [26]. Child overweight/obesity was defined as BAZ > 2 standard deviations (SD), child stunting as HAZ < −2 SD.

Individual-level dietary quality was evaluated with the food-intake data from the 24 h recalls. Simple dietary quality indicators are the food variety score (FVS), the dietary diversity score (DDS), and the healthy eating index (HEI-2015) [19,27,28,29,30,31]. FVS is a simple count of the different food items eaten by the individual during the 24 h recall period. DDS is a count of the number of different food groups eaten, considering a total of nine healthy food groups (Supplementary Table S4). FVS and DDS do not consider the quantities of foods consumed. In contrast, HEI considers the quantities of 13 food components consumed, using a scoring method for each of the components.

A bit more context on the HEI and how it was calculated for the individuals in our sample may be useful. The HEI is a measure of diet quality and evaluates how well an actually observed diet aligns with recommended dietary guidelines. It was based on dietary guidelines for Americans [29]. To our knowledge, the HEI has not been used in an African context before, nor are we aware of similar indexes for Africa or other developing-country regions. To some extent, national dietary guidelines consider food habits, which differ across countries and regions. Hence, using American guidelines in the context of Zambia is not ideal. On the other hand, calculation of the HEI builds on broader food groups, such as fruits, vegetables, whole grains, and dairy, for which recommendations do not differ very much internationally [3,7]. The 13 food components considered in the HEI include adequacy components (e.g., whole fruits, whole grains), where higher quantities consumed by an individual are rated with a higher score, and moderation components (e.g., added sugar, sodium), where higher quantities consumed are rated with a lower score. The 13 components and the scoring scheme are shown in Supplementary Table S4. The scores achieved for all components were added up for each individual. We calculated the HEI for all adults and children in our sample, accounting for all food ingredients consumed. We obtained nutrition facts from food composition tables for Zambia and Kenya [32,33]. For processed and packaged foods, we also recorded ingredients and nutrients based on what was indicated on the packaging. Whenever certain details required for the HEI calculations, such as fatty acid compositions, were not available from local/regional food composition tables or the nutrition facts on the packaging, we used data from the United States Department of Agriculture (USDA) standard reference for the particular food items [34].

In addition to the HEI, other indicators of dietary quality that we used are individual-level intakes of calories, protein, and several micronutrients, namely iron, zinc, and vitamin A. While the human body needs a large number of micronutrients for healthy nutrition, deficiencies in these three micronutrients are particularly common in developing countries and responsible for large health problems among children and adults [3]. Quantities of different food items consumed by individuals were converted to calories and nutrients using food composition tables for Zambia [32]. For food items not included in these tables, we used food composition tables for Kenya [33] or the USDA standard reference [34]. Iron intakes were adjusted for 10% bioavailability as recommended for diets high in plant-based foods [33]. For cooked foods, we considered typical local recipes and nutrient retention values. For processed foods, we considered calories and nutrients as stated on the packaging. We also calculated mean micronutrient adequacy ratios, considering estimated average requirements for individual age and sex groups (Supplementary Table S5).

### 2.3. Main Explanatory Variables

We expect that people’s diets and nutrition are influenced—at least to some extent—by where they purchase their food. Hence, the main explanatory variable of interest in our study is the use of modern retailers for food purchases. Modern retailers include hypermarkets, supermarkets, convenience stores, and fast-food restaurants. For the analysis, we measured the use of modern retailers as the share of the total household food expenditures made in modern retailers. This share was calculated using the 7-day household-level food consumption data and was expressed in percentage terms. For non-users of modern retailers, the share of food expenditures spent in modern retailers was 0%. These were truly observed zeros and not missing values, which makes an important difference in the statistical analysis. For users of modern retailers, the share was greater than 0% and ranged up to a maximum of 100%.

Measuring the use of modern retailers as a continuous variable, as we did here, is a common approach in the literature [19,21,23] and carries more information than a simple dummy variable, because households rarely purchase all of their food in either modern or traditional retailers [13]. Nevertheless, for certain parts of the analysis we also used a simple dummy variable, differentiating between users and non-users of modern retailers, as this allowed interesting subsample comparisons. Households were defined as users of modern retailers (dummy variable takes a value of 1) if they had purchased any of the food items consumed in a modern retailer, and as non-users (dummy variable takes a value of 0) if all of the foods had been obtained from traditional sources (traditional markets, groceries, mom-and-pop-shops, kiosks, own production, etc.).

However, people’s diets and nutrition can also be influenced by various other socioeconomic, demographic, and cultural factors that we needed to control for in regression models in order to evaluate the net effects of modern retailers. We controlled for per capita income, measured in US dollars per year, as income is known to be jointly correlated with the use of modern retailers and with diet and nutrition outcomes [15,16,17,18,19,20,21]. Furthermore, we controlled for other potentially confounding factors such as household size, individual age (years), sex, education (schooling years), health conditions, ethnicity, and religion, among others. A full list of control variables is shown in Supplementary Table S6.

### 2.4. Statistical Analysis

We started the analysis with descriptive statistics by comparing various diet and nutrition indicators between the subsamples of users and non-users of modern retailers. Differences between subsample mean values were tested for statistical significance using t-tests (or Mann–Whitney tests for not-normally distributed variables).

However, simple mean value comparisons can be misleading, as they do not control for potentially confounding factors. To control for confounding factors and estimate the net effects of modern retailer use on diets and nutrition, we estimated regression models of the following type:

(1) $$N_{i}=\alpha +\beta MR_{hi}+\gamma X_{i}+\delta X_{hi}+\varepsilon_{i}$$

where $N_{i}$ is the nutrition status or dietary quality indicator of individual *i* and $MR_{hi}$ is the use of modern retailers for food purchases in household *h* that individual *i* belongs to. As mentioned, we measured $MR_{hi}$ as the share of total household food expenditures made in modern retailers expressed in percentage terms. The estimation coefficient β indicates whether food purchases in modern retailers influence individual diets and nutrition after controlling for other factors. In the estimates, we controlled for individual characteristics ($X_{i}$) and household characteristics ($X_{hi}$). Control variables are explained in more detail in the previous subsection. $\varepsilon_{i}$ is a random error term. We estimated all models separately for adults and children. Models for dietary composition in terms of food processing levels were estimated at the household level.

For outcome variables that were measured with count data (FVS, DDS, HEI), we used Poisson models for estimation. Overweight/obese is a dummy variable, for which we used a probit specification. Several other outcomes are censored variables (calorie and nutrient intakes) for which we used Tobit specifications. For dependent variables that are continuous and normally distributed (expenditure share for foods at different processing levels, BMI, HAZ), we used linear regression models.

As $MR_{hi}$ is likely endogenous (households decide themselves how intensively they use modern retailers), we used a control function (CF) approach with instrumental variables to test and control for possible correlation with the error term (Supplementary Methods). The CF approach is suitable to address issues of endogeneity in linear and non-linear models, so the estimation coefficient β can be cautiously interpreted in a causal way [35]. As a robustness check, we also used Heckman selection models to account for the fact that the modern retailer use variable is censored at zero [36]. We used bootstrapped standard errors clustered at the compound level. All data analyses were performed with the software package Stata/SE 15.1.

## 3. Results

### 3.1. Descriptive Comparisons

Three-quarters of all households in the sample used modern retailers during the 7 days prior to the survey, at least for some of their food purchases. The rest obtained all of the foods consumed from traditional sources. Users of modern retailers spend 59% of their total food expenditures in modern retailers on average. Table 1 shows that users of modern retailers consume lower quantities of vegetables and pulses and higher quantities of meat, dairy, sugar, and beverages than non-users. This comparison points to notable dietary differences between the two groups.

**Figure 1:**
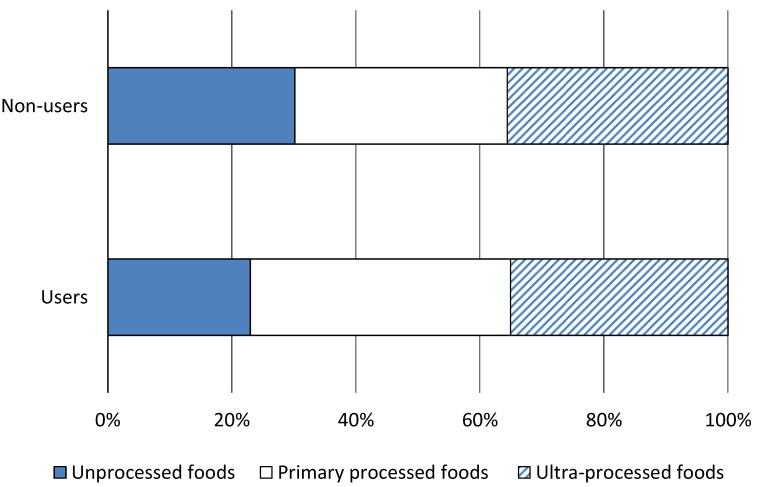
Dietary composition among users and non-users of modern retailers. Average household-level dietary composition is shown in terms of expenditure shares spent on foods at different processing levels. The sample includes 360 households that used and 115 households that did not use modern retailers. Differences in expenditure shares were tested for statistical significance, as shown in Supplementary Table S7.

Figure 1 shows household-level dietary composition in terms of food processing levels among users and non-users of modern retailers. Some notable differences can be observed. Households using modern retailers spend a lower share of their food expenditures on unprocessed foods and a higher share on primary processed foods than non-users of modern retailers. These differences are statistically significant (Supplementary Table S7). However, we do not observe a significant difference for ultra-processed foods. Both users and non-users spend around 35% of their total food expenditures on ultra-processed foods. This is a clear indication that traditional retailers, such as kiosks and mom-and-pop-shops, also sell a lot of ultra-processed foods.

Table 2 shows individual diet and nutrition outcomes with and without modern retailer use. Overweight/obesity is fairly widespread, affecting 40–50% of the adults with no significant differences between the two groups. For children, overweight/obesity rates are much lower at 5–6%. Children are more affected by undernutrition; the prevalence of child stunting is lower among modern retail users than among non-users, even though the difference is not statistically significant.

For the dietary indicators, several significant differences are observed. Adults and children in households using modern retailers have higher food variety scores (FVS), a higher healthy eating index (HEI), and higher vitamin A intakes than their counterparts in households that obtained all of their foods from traditional sources. However, regardless of the differences between the subsamples, dietary quality in urban Zambia is low. An average DDS of around 3 for adults and children is very low, even by developing-country standards [3,6,27]. The average HEI of around 30 is also very low. In the USA, the HEI is in the 50s for people at higher mean income levels and in the 40s for people in the lower income segments [28,29,30,31]. Mean micronutrient adequacy ratios are below 100% for all of our subsamples from urban Zambia (Supplementary Table S6). These patterns clearly point at multiple burdens of malnutrition within the same households and individuals.

The differences in dietary and nutrition outcomes between users and non-users of modern retailers observed are shown in Table 1; Table 2 as well as Figure 1 cannot be interpreted as effects of modern retailers, because the groups also differ in terms of various other characteristics. For instance, users of modern retailers have significantly higher incomes and education levels than non-users (Supplementary Table S6). As explained above, we use regression models with instrumental variables to control for such confounding factors and to be able to draw cautious causal inference.

### 3.2. Effects of Modern Retailers on Dietary Composition in Terms of Processing Levels

Table 3 shows the estimated net effects of using modern retailers on household-level dietary composition in terms of food processing levels. After controlling for household income and other confounding factors, a 10 percentage point increase in the food expenditure share spent in modern retailers decreases the share of unprocessed foods in people’s diets by 0.71 percentage points (column 1), whereas it increases the share of ultra-processed foods by 0.53 percentage points (column 3). The effect on the share of primary processed foods consumed is positive but not statistically significant. As mentioned, users of modern retailers spend 59% of their total food expenditures in modern retail outlets on average. We can use this average and the estimates from Table 3 for some linear extrapolation: moving from not using modern retailers at all (expenditure share of 0%) to the average use intensity among users of 59% would lead to a 4.2 percentage point decrease in unprocessed foods and a 3.1 percentage point increase in ultra-processed foods in people’s diets. Very similar effects are also obtained when estimating the effects of modern retail use with Heckman selection models (Supplementary Table S9).

In spite of its statistical significance, the net effect of modern retailers on the consumption of ultra-processed foods is not very large in magnitude. In order to better understand the relationship between using modern retailers and consuming ultra-processed foods, we reran the same models as shown in Table 3 but only using the subsample of modern retail users (excluding the 0% observations for the modern retailer expenditure share). In these alternative specifications, the coefficient is somewhat larger (Supplementary Table S9), suggesting that increasing the modern retailer use intensity at higher levels has a larger effect on ultra-processed food consumption than starting to use modern retailers at low intensity levels. This interpretation is further supported by the statistically insignificant effect on ultra-processed food consumption in model specifications with the full sample where the use of modern retailers is expressed as a simple dummy variable (Supplementary Table S9).

### 3.3. Effects of Modern Retailers on Nutrition Status

Table 4 shows the estimated net effects of using modern retailers on individual nutrition status. We start by interpreting the results for adults. After controlling for household income and other confounding factors, a 10 percentage point increase in the food expenditure share spent in modern retailers increases adult BMI by 0.12 kg/m^2^ (column 1). Again, we use this estimate for linear extrapolation: moving from not using modern retailers at all (expenditure share of 0%) to the average expenditure share among users (59%) would lead to a 0.71 kg/m^2^ increase in adult BMI, after controlling for income and other relevant factors. As mentioned, a considerable proportion of the adults are already overweight or obese. The risk of adult overweight/obesity further rises through the use of modern retailers: a 10 percentage point increase in the modern retailer expenditure share raises the likelihood of overweight/obesity by 4 percentage points (column 2 of Table 4).

For children, the results are different. Using modern retailers has no significant effect on child BMI-for-age Z-scores (column 3 of Table 4), and the effect on child overweight/obesity is even negative (column 4). At the same time, we observe a statistically significant positive effect on child height (column 5). A 10 percentage point increase in the modern retail expenditure share raises child-for-age Z-scores (HAZ) by 0.26. Hence, moving from not using modern retailers at all (expenditure share of 0%) to the average expenditure share among users (59%) would lead to an increase in HAZ by 1.51 SD, thus reducing the likelihood of child stunting considerably. This points to clearly positive effects of modern retailers on child nutrition. When using Heckman selection models as a robustness check, we also find increasing effects of modern retailers on adult overweight/obesity, whereas for children we find positive effects on child HAZ (Supplementary Table S11).

### 3.4. Effects of Modern Retailers on Dietary Diversity and Nutrient Intakes

**Figure 2:**
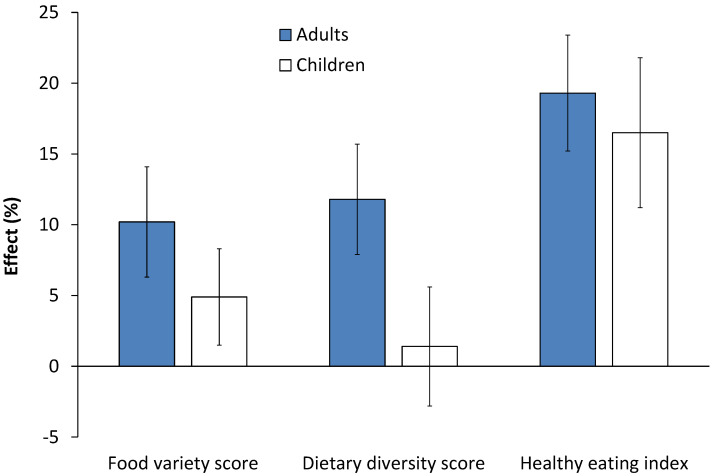
Effects of using modern retailers on dietary diversity. Percentage effects are shown with standard error bars. Use of modern retailers expressed as a dummy variable that takes a value of one if any of the food consumed was purchased in a modern retailer and zero if all of the foods consumed were obtained from traditional sources. Effects were estimated with control function regression models, controlling for income, education, age, and other relevant factors. Models for adults were estimated with 930 individual observations. Models for children were estimated with 499 individual observations. Full model results are shown in Supplementary Tables S12 and S13.

Figure 2 shows effects of modern retailers on adult and child dietary diversity. After controlling for income and other relevant factors, use of modern retailers increases adult FVS and DDS by 10–12%. For children, the point estimates for FVS and DDS are positive but not statistically significant. However, FVS and DDS only count the number of food items and food groups consumed without considering food quantities. When considering intake quantities of different food components through the HEI, the effects are larger and statistically significant. Use of modern retailers increases HEI by 19% and 17% for adults and children, respectively (Figure 2). These effects are particularly due to the higher consumption of meat and dairy among modern retail users. While the HEI remains below recommended levels for most individuals even with these improvements, using modern retailers seems to move dietary quality at least in the right direction. In any case, we use FVS, DDS, and HEI only to get a first impression of the effects of modern retailers on dietary quality. More detailed insights can be gained from the data on actual nutrient intakes.

**Figure 3:**
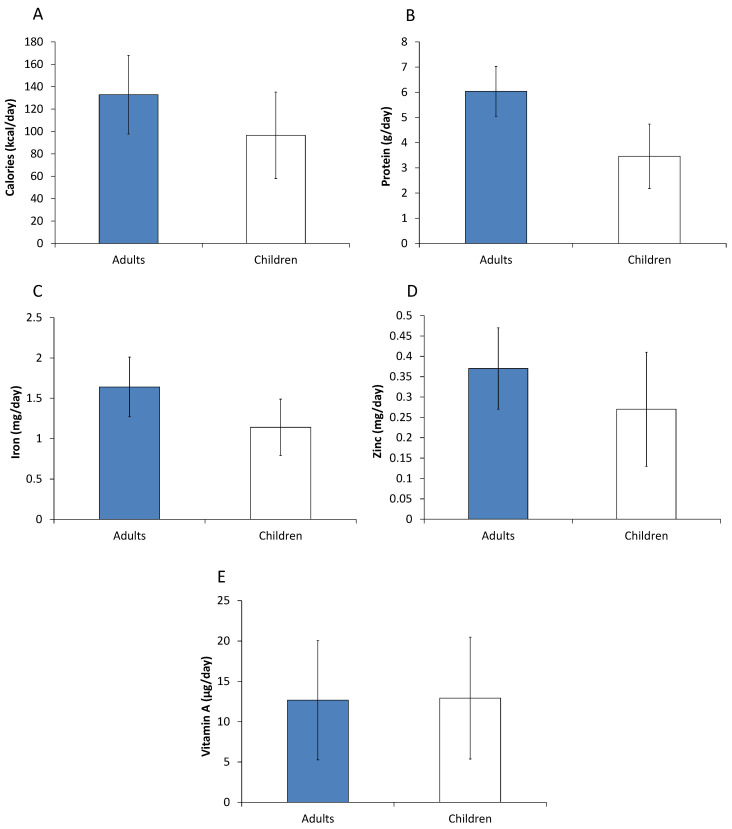
Effects of using modern retailers on calorie and nutrient intakes. Effects of a 10 percentage point increase in the household food expenditure share spent in modern retailers are shown with standard errors bars. Effects were estimated with control function regression models, controlling for income, education, age, and other relevant factors. Models for adults were estimated with 930 individual observations. Models for children were estimated with 499 individual observations. Full model results are shown in Supplementary Tables S14 and S15. (**A**) Effects on calorie intake in kcal/day. (**B**) Effects on protein intake in g/day. (**C**) Effects on iron intake in mg/day. (**D**) Effects on zinc intakes in mg/day. (**E**) Effects on vitamin A intakes in µg of retinol equivalents per day.

Figure 3 shows effects of modern retailers on calorie and nutrient intakes. All effects are positive and statistically significant. After controlling for other factors, a 10 percentage point increase in the modern retail expenditure share raises calorie intakes by 133 and 97 kcal/day for adults and children, respectively. For adults, the additional calorie intake probably also explains the increase in BMI through using modern retailers, as shown above.

Modern retailers also have positive effects on nutrient intakes (Figure 3). A 10 percentage point increase in the modern retail expenditure share augments adult and child protein, iron, and zinc intakes by 5–7%; for vitamin A, the increase is about 3% for both adults and children. These are sizeable effects, especially when considering that users of modern retailers spend 59% of their total food expenditures in modern retail outlets. The estimated net effects of using modern retailers lift a considerable share of individuals above the average requirements for the different micronutrients. This is also reflected in positive and significant effects on mean micronutrient adequacy ratios, as shown in Supplementary Tables S16 and S17. These findings clearly underline that modern retailers improve adult and child micronutrient nutrition.

Most previous studies on the effects of modern retailers have focused on supermarkets only, not considering hypermarkets, convenience stores, and fast-food restaurants. In order to test whether our results change when only considering supermarkets, we reran the calorie and nutrient intake models with a modified modern retailer variable that only captures the supermarket food expenditure share. These alternative estimates are similar to those shown in Figure 3, only the effect sizes are somewhat smaller (Supplementary Table S18), as one would expect. We infer that the different types of modern retailers have similar effects.

**Figure 4:**
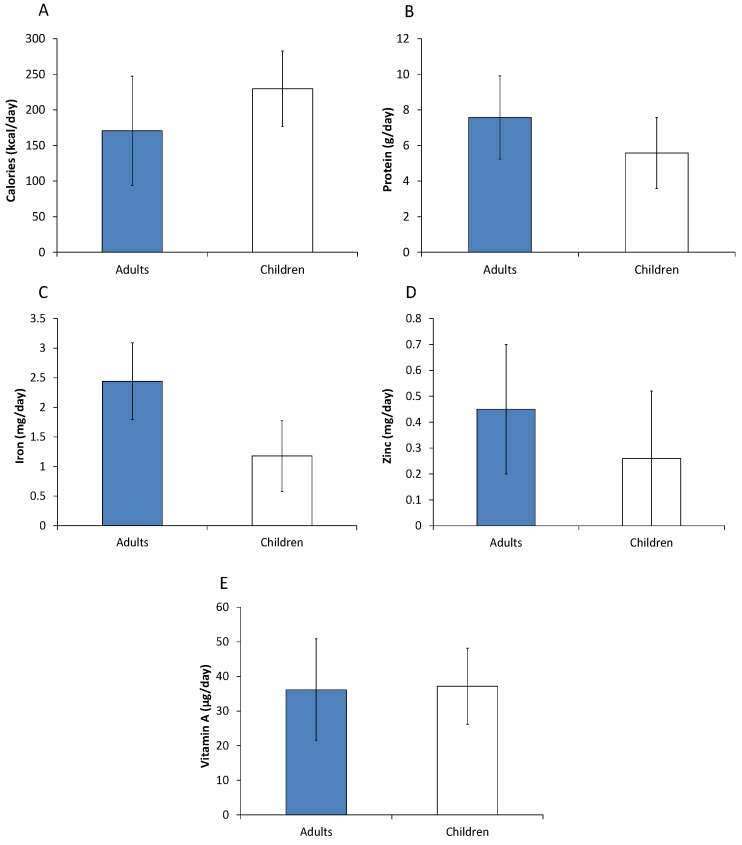
Effects of using modern retailers on calorie and nutrient intakes among poor households. Effects of a 10 percentage point increase in the household food expenditure share spent in modern retailers are shown with standard errors bars. Effects were estimated with control function regression models, controlling for income, education, age, and other relevant factors. Poor households are defined as those with incomes less than $1.90 per capita and day. Models for adults were estimated with 226 individual observations. Models for children were estimated with 175 individual observations. Detailed model results are shown in Supplementary Table S19. (**A**) Effects on calorie intake in kcal/day. (**B**) Effects on protein intake in g/day. (**C**) Effects on iron intake in mg/day. (**D**) Effects on zinc intakes in mg/day. (**E**) Effects on vitamin A intakes in µg of retinol equivalents per day.

In additional analyses, we looked specifically at effects on individuals living below the international poverty line of 1.90 US dollars per day. About 24% of the adults and 35% of the children in the sample are poor according to this definition. Interestingly, most of the effects of modern retailers on calorie and nutrient intakes are larger for poor individuals (Figure 4) than for the full adult and child samples (Figure 3). For vitamin A, the positive intake effects on poor individuals are almost three times larger. This is a welcome finding, as it implies that poor people benefit over-proportionally from access to modern retailers.

Finally, we disaggregated the adult and child samples by sex. While the estimates are generally less efficient (due to the smaller subsamples), positive calorie and nutrient intake effects are observed for male and female adults and children (Supplementary Table S20). The estimated effects of modern retailers on micronutrient intakes are somewhat larger for girls than for boys.

## 4. Discussion

### 4.1. Discussion of Main Findings and Policy Implications

In Zambia and many other developing countries, food environments are changing rapidly, with modern retailers gaining in importance. Most households use both modern and traditional retailers for their food purchases, but the share of the food budget spent in modern retail outlets is rising [13,25]. According to our data, the average household in Lusaka spends about 42% of its food budget for purchases in modern retail outlets. Excluding those that only use traditional sources, the modern retail share increases to 59%. Changing food environments can influence people’s dietary choices and nutrition. Modern retailers tend to sell more processed foods than traditional markets. Moreover, there are differences in terms of food variety, prices, packaging sizes, and shopping atmosphere. Previous studies suggested that modern retailers increase the consumption of calories from processed foods and therefore contribute to overweight, obesity, and related chronic diseases [15,17,19,20]. These studies mostly looked at adult populations. Very few studies have analyzed effects of modern retailers on child nutrition, and those that did found mixed results [16,21]. One shortcoming of all existing studies is that they did not collect individual-level food-intake data. Individual-level data are important to analyze effects of modern retailers on dietary quality and better understand the mechanisms behind the nutrition impacts.

In this article, we evaluated dietary and nutrition effects of modern retailers in Lusaka using individual-level food-intake and anthropometric data. We estimated regression models to control for income and other factors that may also influence nutrition and used instrumental variables to test for unobserved heterogeneity and reduce related bias. Our results suggest that the use of modern retailers is positively associated with BMI and the likelihood of being overweight and obese among adults. This is consistent with earlier results for adult populations in Guatemala and Kenya [15,17,19]. However, for children we did not find a significant relationship between the use of modern retailers and BMI-for-age Z-scores. Instead, we found that the use of modern retailers is positively associated with child height. This is consistent with recent results for children in urban Kenya [21]. Gains in child height point at likely improvements in dietary quality and nutrient intakes, even though—due to data limitations—this had not been analyzed in previous studies.

Analysis of our individual-level dietary data confirms that the use of modern retailers increases dietary intakes for both adults and children. First, modern retailers contribute to higher calorie intakes, resulting primarily from more consumption of sugar, meat, and dairy products. Second, especially the increase in the consumption of animal source foods also contributes to higher intakes of protein and micronutrients, such as iron, zinc, and vitamin A. These dietary effects are observed for both adults and children. For adults, this implies negative and positive nutrition effects at the same time: the growth of modern retailers is associated with a rise in overweight/obesity and a reduction in micronutrient deficiencies among adults. For children that have not yet reached their final body height, the mechanisms are different. Increases in calorie, protein, and micronutrient intakes contribute to linear growth, especially in situations where child stunting is still commonplace. Recent research with data from a large number of developing countries showed that consumption of animal source foods in particular is positively associated with child linear growth [37]. This is especially true in situations where regular access to sufficient quantities of plant-based proteins and micronutrients is difficult.

Beyond nutrient intakes, our results showed that the use of modern retailers contributes to a shift in household dietary composition away from unprocessed foods towards ultra-processed foods. This is consistent with findings from other developing countries [14,15,17,18,19,23]. Ultra-processed foods are often considered less healthy than unprocessed foods. However, differentiation is important. Ultra-processed foods with high sugar, fat, and salt, and low micronutrient contents are unhealthy and known to contribute to the obesity pandemic [1,4,8]. Cases in point are various convenience foods, as well as snacks and soft drinks. However, not all processed and ultra-processed foods are unhealthy. Processing can increase the hygiene and shelf-life of foods and therefore make nutritious, perishable products more accessible to consumers. Meat and dairy are good examples. Many poor households rarely buy fresh meat and milk, as these are expensive and highly perishable. Hence, access to processed versions with a longer shelf-life can increase the consumption of nutritious foods. This also explains why the effects of modern retailers on micronutrient intakes of individuals from poor households are particularly large, as we found when looking separately at the subsample of people living below the international poverty line.

A few policy implications emerge from these results. The growth of modern retailers influences people’s diets and nutrition, and the effects can be positive and negative. The positive effects on child nutrition and dietary quality of both children and adults are not yet widely appreciated and speak in favor of supporting further modernization of food retail environments. On the other hand, the effect of increasing adult overweight and obesity is undesirable. Regulatory policies that can help to make food environments healthier would be useful. Possible policy interventions include the regulation of advertisement and promotional campaigns for unhealthy foods, such as snacks, soft drinks, and ready-made dishes rich in sugar, salt, and fat. Food policies to promote more healthy diets could also involve improved designs of mandatory and voluntary health labels, regulation of portion and packaging sizes, taxes on particularly unhealthy foods and beverages, subsidies for particularly healthy products, and other types of incentives for retailers to offer more healthy foods [1,3,5].

### 4.2. Study Limitations

Our study has a few limitations that should be discussed. First, we used a variety of dietary quality indicators, each of which has certain drawbacks. Food variety scores and dietary diversity scores are simple count measures that do not consider food processing levels and quantities consumed. The healthy eating index accounts for quantities consumed but was developed based on dietary guidelines for consumers in the USA, not in Africa. Dietary habits and the seasonal availability of foods vary geographically, so that an index developed for American consumers is not ideal in an African context. The other dietary indicators also have their limitations. We used a common classification to categorize foods by their processing level, but the three categories used (unprocessed, primary processed, ultra-processed) are imperfect proxies of the (un)healthiness of food items. The most direct measures of dietary quality that we used were individual-level nutrient intakes, but we only focused on a few key nutrients and did not analyze the whole spectrum of nutrients required for healthy nutrition. Moreover, our food-intake data were collected through a single 24 h dietary recall per respondent, so individual-level dietary variation is probably underestimated. Hence, while the suite of indicators used here provides a useful picture, results for individual indicators should be interpreted with caution.

A second limitation relates to the fact that we used cross-section observational data, which has its limitations for drawing rigorous causal inference. We used regression models to control for observed heterogeneity and instrumental variables to test and control for unobserved heterogeneity, so some cautious causal inference should be in order. Nevertheless, instrumental variables are rarely perfect, implying that a certain degree of endogeneity bias may remain. Hence, while the identified associations are consistent and plausible, overinterpretation in terms of strong causality should be avoided. Follow-up research with panel data or experimental approaches would be useful to verify causal relationships.

A third limitation relates to representativeness of the sample. We tried hard to obtain a sample that covers all socioeconomic strata of households living in Lusaka by selecting city compounds with low, middle, and high mean income levels. We expect that the sample of households is fairly representative, but in the sample of individuals, adult males are somewhat underrepresented. The reason is that adult males living in the sample households were sometimes absent during the scheduled interview times, even when we tried to reach them during a second visit. This explains why we have more female adults than male adults in the sample of individuals. However, the quality of the household-level information was likely not affected by the absence of males, as female adults are mostly the ones responsible for food purchases and food preparation in the household.

A fourth limitation relates to external validity. Our results are specific for Lusaka in Zambia and should not be generalized. It is likely that the diet and nutrition effects of modern retailers will be similar in situations where households are relatively poor, child stunting is widespread, and people only have limited or irregular access to healthy foods from traditional markets. This is probably the case in many parts of Africa, as recent research with data from Kenya also suggests [17,19,21,23]. However, the effects of modern retailers may be different in situations where consumers are richer, micronutrient deficiencies are not a big problem, and child undernutrition rates are low. For instance, one study with data from Indonesia suggested that the use of modern retailers contributes to child overweight in high-income households [16]. More research is needed to better understand the diet and nutrition effects of changing food environments in different geographical and socioeconomic contexts.

## 5. Conclusions

Food environments in developing countries are changing rapidly, with modern retailers—such as supermarkets and convenience stores—increasingly gaining in importance. In this article, we analyzed effects of using modern retailers on people’s dietary quality and nutrition with data from adults and children in Lusaka, the capital city of Zambia. We used regression models to control for socioeconomic status and other possible confounding factors to the extent possible. We found that the use of modern retailers, measured in terms of the food expenditure share spent in supermarkets and other modern retail outlets, is positively associated with the consumption of ultra-processed foods and with overweight in adults. For children, we did not find a significant effect of using modern retailers on weight; instead, we found a positive association with body height. Furthermore, the data showed that the use of modern retailers is positively associated with dietary diversity, as well as with calorie, protein, and micronutrient intakes among both adults and children. Effects on protein and micronutrient intakes are channeled primarily through higher consumption of meat and dairy products. The results demonstrate that modern retailers can influence people’s diets and nutrition in positive and negative ways. Evidence-based and differentiated regulatory policies will be needed to create food environments that promote healthy food choices and nutrition in Africa and beyond.

## Figures and Tables

**Table 1:** Per capita food intake of adults and children using and not using modern retailers.

Food Intake (g/day)	Adults (≥18 Years)		Children (<18 Years)	
	Modern Retailers		Modern Retailers	
	Users	Non-Users	Users	Non-Users
	(*n* = 713)	(*n* = 217)	(*n* = 358)	(*n* = 141)
Cereals and tubers	569.18	576.57	427.77	396.39
	(288.83)	(298.11)	(232.81)	(225.30)
Pulses	12.36 ***	24.07	9.18 **	22.77
	(39.41)	(89.14)	(25.95)	(100.90)
Vegetables	47.33 ***	78.05	31.82 ***	58.28
	(71.80)	(123.14)	(63.30)	(89.26)
Fruits	3.30	3.04	1.19 ***	4.09
	(18.94)	(21.75)	(5.47)	(17.41)
Meat	36.66 ***	22.64	26.82 ***	14.95
	(43.80)	(47.43)	(31.40)	(46.14)
Dairy products	19.76 **	7.85	22.22 *	12.41
	(76.96)	(47.41)	(59.43)	(58.94)
Eggs	7.69	10.63	5.59 *	10.80
	(24.07)	(46.93)	(19.85)	(42.16)
Fish	19.33	23.29	11.79	14.40
	(55.96)	(63.53)	(38.55)	(47.60)
Sugar, beverages	171.80 ***	124.83	140.79 **	101.25
	(196.37)	(173.95)	(171.75)	(130.71)
Oils and fats	0.65	0.59	0.56	0.59
	(2.28)	(1.14)	(2.95)	(1.08)

Mean values are shown with standard deviations in parentheses. Mean differences between users and non-users of modern retailers were tested for statistical significance. * *p* < 0.10, ** *p* < 0.05, *** *p* < 0.01. *n*, number of observations.

**Table 2:** Nutrition and dietary indicators for adults and children using and not using modern retailers.

		Adults (≥18 Years)		Children (<18 Years)	
Variables	Units	Modern Retailers		Modern Retailers	
		Users	Non-Users	Users	Non-Users
		(*n* = 713)	(*n* = 217)	(*n* = 358)	(*n* = 141)
Body mass index (BMI)	kg/m^2^ or BMI-for-age Z score	25.86	25.51	0.05	–0.18
		(4.88)	(5.65)	(1.45)	(1.72)
Overweight or obese	1 if BMI ≥ 25 or BAZ > 2 SD	0.50	0.44	0.05	0.06
		(0.50)	(0.50)	(0.22)	(0.24)
Height-for-age Z score (HAZ)	Z score	NA	NA	–0.51	–0.72
				(1.51)	(1.59)
Stunting	1 if HAZ < –2 SD	NA	NA	0.15	0.21
				(0.36)	(0.41)
Food variety score (FVS)	Score; range (0–18)	6.64 **	6.26	6.69 **	6.28
		(1.85)	(2.11)	(1.94)	(1.49)
Dietary diversity score (DDS)	Score; range (0–9)	3.23	3.12	3.02	3.08
		(1.02)	(1.00)	(1.00)	(1.00)
Healthy eating index (HEI)	Score; range (0–100)	32.58 ***	29.77	31.59 ***	28.41
		(10.12)	(10.94)	(10.88)	(10.73)
Calorie intake	kcal/day	2653.11 **	2457.08	2006.76	1964.00
		(1161.83)	(985.42)	(936.00)	(969.40)
Protein intake	g/day	81.28	80.96	60.44	60.62
		(35.49)	(39.30)	(33.10)	(34.37)
Iron intake	mg/day	23.88	24.61	17.05	18.41
		(11.71)	(12.84)	(9.50)	(12.19)
Zinc intake	mg/day	7.59	7.64	5.36	5.47
		(5.45)	(6.19)	(3.10)	(5.44)
Vitamin A intake	µg retinol/day	525.83 ***	409.33	473.48**	380.22
		(499.93)	(454.70)	(487.48)	(428.93)

Mean values are shown with standard deviations in parentheses. Mean differences between users and non-users of modern retailers were tested for statistical significance. ** *p* < 0.05, *** *p* < 0.01. *n*, number of observations; NA, not applicable. SD, standard deviation. Additional variables are shown in Supplementary Table S6.

**Table 3:** Effects of using modern retailers on dietary composition in terms of food processing levels.

	Unprocessed Foods	Primary Processed Foods	Ultra-Processed Foods
	(Expenditure Share, %)	(Expenditure Share, %)	(Expenditure Share, %)
	(1)	(2)	(3)
Modern retail use	−0.071 **	0.018	0.053 *
(expenditure share, %)	(0.027)	(0.023)	(0.025)
Control variables	Yes	Yes	Yes
Joint F-statistic	9 ***	12 ***	5 **
*n*	475	475	475

Marginal effects from ordinary least squares regressions are shown with robust standard errors clustered at compound level in parentheses. The null hypothesis of modern retailer use being exogenous could not be rejected. Full model results with all control variables are shown in Supplementary Table S8. * *p* < 0.10, ** *p* < 0.05, *** *p* < 0.01. *n*, number of household observations.

**Table 4:** Effects of using modern retailers on nutrition status.

	Adults (≥18 Years)			Children (<18 Years)	
	BMI (kg/m^2^)	Overweight/Obese (1,0)	BAZ (Z-Score)	Overweight/Obese (1,0)	HAZ (Z-Score)
	(1)	(2)	(3)	(4)	(5)
Modern retail use	0.012 **	0.004 ***	−0.011	−0.016 **	0.026 ***
(expenditure share, %)	(0.005)	(0.002)	(0.008)	(0.007)	(0.008)
Control variables	Yes	Yes	Yes	Yes	Yes
Joint F-statistic/Wald χ2	761 ***	862 ***	66 ***	35 ***	117 ***
n	863	863	458	458	472

Marginal effects are shown with robust, bootstrapped standard errors clustered at compound level in parentheses. For the adult sample, the null hypothesis of modern retailer use being exogenous could not be rejected, so standard ordinary least squares and probit estimates are shown. For the child sample, the null hypothesis of exogeneity was rejected, so control function estimates are shown. Full model results with all control variables are shown in Supplementary Table S10. ** *p* < 0.05, *** *p* < 0.01. BAZ, BMI-for-age Z-score; BMI, body mass index; HAZ, height-for-age Z-score; *n*, number of individual observations.

## Supplementary Materials

The following are available online at https://www.mdpi.com/2072-6643/12/6/1714/s1, Figure S1: Map of Lusaka City with sampled compounds/sections and households, Table S1: List of main shopping malls with modern retailers in Lusaka City in 2018, Table S2: Distribution of sampled individuals in Lusaka City, Table S3: Food processing levels by food groups and items, Table S4: Food groups and components used for construction of dietary quality indicators, Table S5: Estimated average requirements of calories and nutrients by sex and age cohort, Table S6: Additional descriptive statistics for users and non-users of modern retailers, Table S7: Household expenditure shares for foods at different processing levels, Table S8: Effects of using modern retailers on dietary composition in terms of food processing levels (full model results for Table 3), Table S9: Effects of modern retailers on dietary composition in terms of food processing levels (alternative model specifications), Table S10: Effects of modern retailers on nutritional status (full model results for Table 4), Table S11: Effects of modern retailers on nutritional status (Heckman selection model results), Table S12: Effects of modern retailers on adult dietary diversity (full model results for Figure 2), Table S13: Effects of modern retailers on child dietary diversity (full model results for Figure 2), Table S14: Effects of modern retailers on adult calorie and nutrient intakes (full model results for Figure 3), Table S15: Effects of modern retailers on child calorie and nutrient intakes (full model results for Figure 3), Table S16: Effects of modern retailers on adult micronutrient adequacy ratios, Table S17: Effects of modern retailers on child micronutrient adequacy ratios, Table S18: Effects of supermarkets on child and adult calorie and nutrient intakes, Table S19: Effects of modern retailers on calorie and nutrient intakes of individuals from poor households (model results for Figure 4), Table S20: Effects of modern retailers on calorie and nutrient intakes disaggregated by sex, Table S21: First-stage estimation results on food purchases in modern retailers, Table S22: Falsification test for instrument validity (Tobit estimates), Supplementary Data: Data and Codes.

## References

[1] Swinburn B., Kraak I.V., Allender S., Atkins V.J., Baker I.P., Bogard J.R., Brinsden H., Calvillo A., De Schutter O., Devarajan R. (2019). The global syndemic of obesity, undernutrition, and climate change: The Lancet Commission report. Lancet.

[2] FAO (2019). The State of Food Security and Nutrition in the World 2019.

[3] Development Initiatives (2018). Global Nutrition Report 2018.

[4] Popkin B.M., Corvalan C., Grummer-Strawn L.M. (2019). Dynamics of the double burden of malnutrition and the changing nutrition reality. Lancet.

[5] Hawkes C., Ruel M.T., Salm L., Sinclair B., Branca F. (2020). Double-duty actions: Seizing programme and policy opportunities to address malnutrition in all its forms. Lancet.

[6] Fongar A., Gödecke T., Qaim M. (2019). Various forms of double burden of malnutrition problems exist in rural Kenya. BMC Public Health.

[7] HLPE (2017). Nutrition and Food Systems.

[8] Popkin B.M. (2017). Relationship between shifts in food system dynamics and acceleration of the global nutrition transition. Nutr. Rev..

[9] Reardon T., Timmer C.P. (2014). Five inter-linked transformations in the Asian agrifood economy: Food security implications. Glob. Food Secur..

[10] Qaim M. (2016). Globalisation of agrifood systems and sustainable nutrition. Proc. Nutr. Soc..

[11] Hawkes C. (2008). Dietary implications of supermarket development: A global perspective. Dev. Policy Rev..

[12] Tessier S., Traissac P., Maire B., Bricas N., Eymard-Duvernay S., El Ati J., Delpeuch F. (2008). Regular users of supermarkets in greater Tunis have a slightly improved diet quality. J. Nutr..

[13] Khonje M.G., Qaim M. (2019). Modernization of African food retailing and (un)healthy food consumption. Sustainability.

[14] Popkin B.M., Reardon T. (2018). Obesity and the food system transformation in Latin America. Obes. Rev..

[15] Asfaw A. (2008). Does supermarket purchase affect the dietary practices of households? Some empirical evidence from Guatemala. Dev. Policy Rev..

[16] Umberger W.J., He X., Minot N., Toiba H. (2015). Examining the relationship between the use of supermarkets and over-nutrition in Indonesia. Am. J. Agric. Econ..

[17] Kimenju S.C., Rischke R., Klasen S., Qaim M. (2015). Do supermarkets contribute to the obesity pandemic in developing countries?. Public Health Nutr..

[18] Kelly M., Seubsman S.A., Banwell C., Dixon J., Sleigh A.C. (2014). Thailand’s food retail transition: Supermarket and fresh market effects on diet quality and health. Br. Food J..

[19] Demmler K.M., Ecker O., Qaim M. (2018). Supermarket shopping and nutritional outcomes: A panel data analysis for urban Kenya. World Dev..

[20] Demmler K.M., Klasen S., Nzuma J.M., Qaim M. (2017). Supermarket purchase contributes to nutrition-related non-communicable diseases in urban Kenya. PLoS ONE.

[21] Debela B.L., Demmler K.M., Klasen S., Qaim M. (2019). Supermarket food purchases and child nutrition in Kenya. Glob. Food Secur..

[22] Ruel M., Alderman H. (2013). Nutrition-sensitive interventions and programmes: How can they help to accelerate progress in improving maternal and child nutrition?. Lancet.

[23] Rischke R., Kimenju S.C., Klasen S., Qaim M. (2015). Supermarkets and food consumption patterns: The case of small towns in Kenya. Food Policy.

[24] Rupa J.A., Umberger W.J., Zeng D. (2019). Does food market modernisation lead to improved dietary diversity and diet quality for urban Vietnamese households?. Aust. J. Agric. Resour. Econ..

[25] Harris J., Chisanga B., Drimie S., Kennedy G. (2019). Nutrition transition in Zambia: Changing food supply, food prices, household consumption, diet and nutrition outcomes. Food Secur..

[26] WHO (2006). Multicentre Growth Reference Study Group. WHO Child. Growth Standards: Length/Height-for-Age, Weight-for-Age, Weight-for-Length, Weight-for-Height and Body Mass Index-for-Age: Methods and Development.

[27] Steyn N.P., Nel J.H., Nantel G., Kennedy G., Labadarios D. (2006). Food variety and dietary diversity scores in children: Are they good indicators of dietary adequacy?. Public Health Nutr..

[28] Beatty T., Lin B.H., Smith T.A. (2014). Is diet quality improving? Distributional changes in the United States, 1989–2008. Am. J. Agric. Econ..

[29] Schap T., Kuczynski K., Hiza H. (2017). Healthy eating index—Beyond the score. J. Acad. Nutr. Diet..

[30] Smith T.A. (2016). Do school food programs improve child dietary quality?. Am. J. Agric. Econ..

[31] Whiteman E.D., Chrisinger B.W., Hillier A. (2018). Diet quality over the monthly supplemental nutrition assistance program cycle. Am. J. Prev. Med..

[32] Nyirenda D.B., Musukwa M., Habulembe R., Shindano J. (2009). Zambia Food Composition Tables.

[33] FAO and Government of Kenya (2018). Kenya Food Composition Tables.

[34] U.S. Department of Agriculture, Agricultural Research Service FoodData Central. http://fdc.nal.usda.gov.

[35] Wooldridge J.M. (2015). Control function methods in applied econometrics. J. Hum. Resour..

[36] Wooldridge J.M. (2010). Econometric Analysis of Cross-Section and Panel Data.

[37] Headey D., Hirvonen K., Hoddinott J. (2018). Animal sourced foods and child stunting. Am. J. Agric. Econ..

