# Supplementary material for: Effects of Modern Food Retailers on Adult and Child Diets and Nutrition
Source: Nutrients. 2020 Jun 8;12(6):1714. doi: 10.3390/nu12061714 (PMC7353018; doi:10.3390/nu12061714)
Supplement: Supplementary file 1 [file nutrients-12-01714-s001.pdf]

SUPPLEMENTARY MATERIALS

Effects of Modern Food Retailers on Adult and Child Diets and Nutrition

Supplementary Methods

Sampling Design

We surveyed a total of 475 households from several compounds/sections in Lusaka City using a two-stage sampling procedure. At the first stage, we purposively selected 14 compounds/sections as primary sampling units based on population distributions and information from the Lusaka City Council on mean income levels in the different compounds/sections. To ensure that the sample is representative and covers a wide variation of socioeconomic situations, we selected four compounds/sections (Avondole, Chalala, Kabulonga, and Woodlands) with high mean income levels, four compounds/sections (Chelston, Chilenje, Kabwata, and PHI) with middle mean income levels, and six compounds/sections (Chawama, Chazanga, Gardens, Kalingalinga, Kaunda Square, and Ng'ombe) with low mean income levels (Supplementary Figure S1). At the second stage, depending on the size of the compound/section, we randomly sampled about 35 households from each compound/section for study participation.

Measuring Micronutrient Adequacy

We calculated nutrient adequacy ratios for iron, zinc, and vitamin A. The micronutrient adequacy ratio was calculated as the individual micronutrient intake divided by the estimated average requirements for the respective age and sex group, expressed as a percentage based on the procedure described by Steyn and colleagues [1]. The estimated average requirements used are shown in Supplementary Table S5. We calculated the mean adequacy ratio by summing the micronutrient adequacy ratios (truncated at 100%) for the three micronutrients and dividing by three [1].

Control Function Approach

In the regression models, the main explanatory variable of interest ("treatment" variable) is food purchases in modern retailers (MR_{hi}). What type of retailers to use for food purchases is a household-level decision that may depend on various factors not all of which we can observe and control for. If unobserved factors are jointly correlated with MR_{hi} and with the dietary and nutrition outcome variables, the coefficient estimate β may be biased.

We used a control function (CF) approach with instrumental variables (IV) to test and correct for such endogeneity bias [2,3]. In comparison to standard IV regressions, the CF estimator is more efficient and can

also be used to control for unobserved heterogeneity in non-linear models, such as probit and Poisson models [3]. The CF was estimated as a two-stage model, whereby the first-stage regression was represented as:

$$MR_{hi} = \theta_0 + \theta_1 X_i + \theta_2 X_{hi} + \theta_3 Z_{hi} + \mu_i$$

where Z_{hi} is a vector of instrumental variables, and μ_i is a random error term. The other variables were defined as in the main paper. The second-stage model was the regression equation shown in the main paper with the individual diet and nutrition outcomes as dependent variable but including the residuals from the first-stage model as an additional regressor. If the residual term was statistically insignificant in the second-stage regression, the null hypothesis of no endogeneity bias could not be rejected, so the CF approach was not required; in that case, the regular one-stage models led to unbiased and more efficient estimates. However, if the residual term turned out statistically significant, the CF approach was preferred to control for endogeneity bias.

Instrumental Variables

We used three instrumental variables for the CF models. First, distance from each household to the closest shopping mall, which was calculated using global positioning system (GPS) data collected during the survey. GPS-based data to measure distance to modern retailers were also used as instruments in several other studies [4–7]. Second, whether or not the household felt that modern retailers sell food of higher quality than traditional retailers, which was also used by Umberger et al. [4]. Third, the number of visits to a shopping mall of the household's closest neighbor in the sample. Beyond distance and accessibility, the neighbor's behavior may capture influence through local social networks [8]. Tests for instrument validity are discussed below.

All three instruments are significantly correlated with the household's own use of modern retailers, MR_{hi} (Supplementary Table S21). As expected, distance to the closest shopping mall is negatively correlated, while the other two instruments are positively correlated with the food expenditure share spent in modern retailers. Moreover, the Wald test for the joint significance of the three instruments is statistically significant at the 1% level for both adults and children (Supplementary Table S21). This underlines that the instruments are relevant. A second important criterion for validity is that the instruments do not affect the dietary and nutrition outcome variables directly, other than through own use of modern retailers. We performed a simple falsification test following Di Falco et al. [9]. Results show that the three instruments are jointly insignificant in all models with dietary and nutrition outcomes as dependent variables (Supplementary Table S22). Hence, we conclude that the instruments are valid.

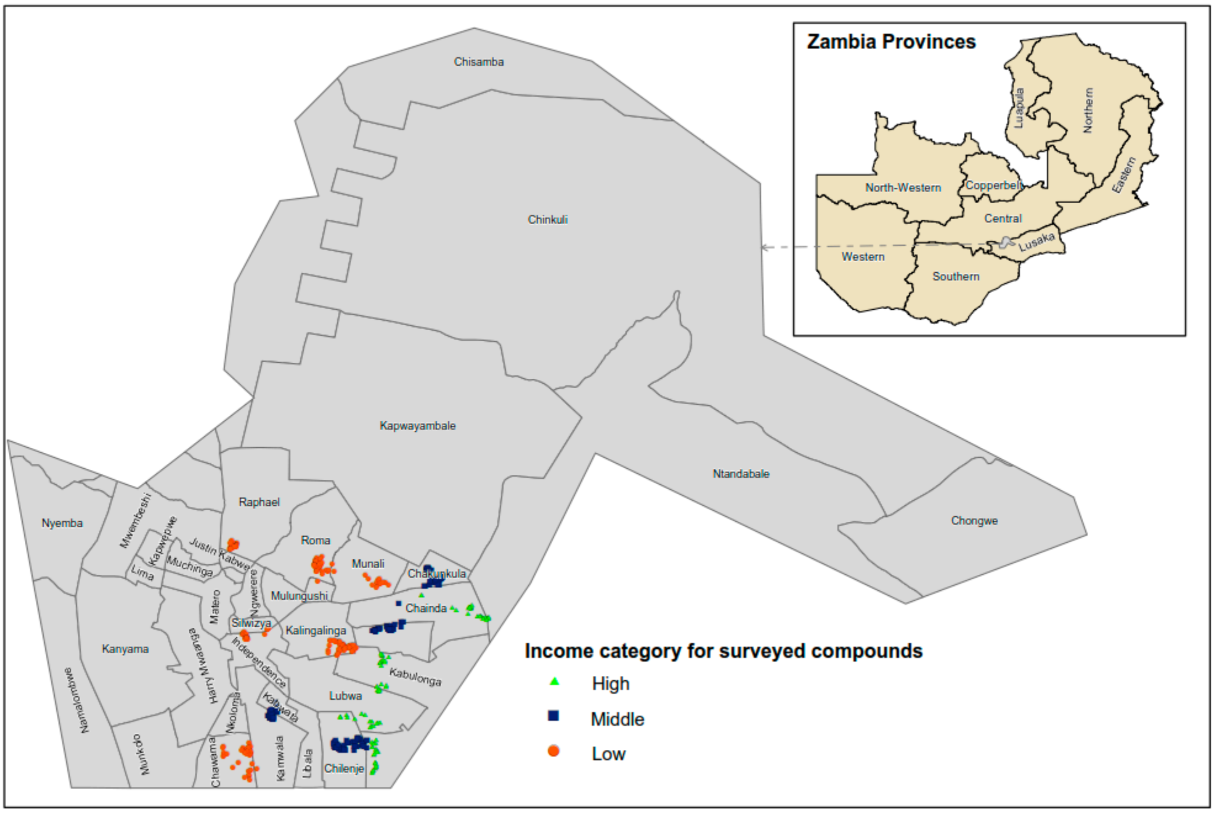

Figure S1. Map of Lusaka City with sampled compounds/sections and households

Table S1. List of main shopping malls with modern retailers in Lusaka City in 2018

No.	Name of Shopping Mall	Location and Surrounding Compounds/Sections	Modern Retailers: Hypermarkets, Supermarkets (Fast-food Restaurants in Parentheses)
1	Arcades	Roma, University of Zambia (UNZA)	Spurs
2	Cairo	Central Lusaka	Shoprite (Food Fayre, Hungry Lion, Machachos)
3	Chawama	Chawama, John Haward, Kuku	Spur
4	Chazanga Shoprite	Chazanga, SOS	Shoprite
5	Chilenje Shoprite	Chalala, Chilenje, Woodlands	Choppies, Shoprite (Debonairs Pizza, MM Chickens, Naaz)
6	Choppies Complex	Kabulonga, Sundel, Zamtel Flats	Choppies
7	Cosmopolitan	Chawama, John Howard, Jon-Lengi, Makeni, Misisi	Game Stores, Shoprite (Chicken Inn, Galito's, Hungry Lion, Mochachos, Pizza Hut)
8	Cross Roads	Cross Road, Kabulonga, Nyumba Yanga, Sundel	Spurs (Gigibonta, Major Meat)
9	Down Town	Chibolya, Jon-Lengi, Kabwata, Kamwala, Misisi	Spurs (Big Bite, Debonairs Pizza, Down Town Foods)
10	East Park	Childley, Kalingalinga, Kalundu, Ng'ombe, Roma, UNZA	Food Lover's, PicknPay (Fishaways, Gigibonta, GoatnChips, Hungry Lion, KEG, Pizza Hut)
11	Embassy	Chawama, Jon-Lengi, Makeni, Misisi	Embassy, Spurs (Papas, Piatto, Zorbas)
12	Garden City	Avondole, Chelston	Food Lover's, PicknPay (Bushman, Foodano)
13	Kabulonga and Melissa	Kabulonga	Melissa, PicknPay (Debonairs Pizza, KFC, Nando's, Subway)
14	Levy Junction	Central Lusaka, Chilulu, Evelyn Home College, Gardens, Nippa, North Mead, Roads Park, Thorn Park	Food Lover's, PicknPay (Chicken Inn, Hungry Lion, KFC, Pizza Inn, Wimpy)
15	Makeni	Chawama, Jon-Lengi, Makeni, Misisi	Food Lover's, PicknPay (Debonairs Pizza, KFC, Nando's)
16	Mama Betty Foxydale	Ngo'mbe, Roma	Spur (Debonairs Pizza, Gigibonta)
17	Manda Hill	Central Lusaka, Chilulu, Gardens, Longacres, Olympia, Roads Park	Shoprite, Game Stores (Bread Café, Debonairs Pizza, Galito's, Hungry Lion, Mugg and Bean, My Asia, Nando's, Pizza Inn, Steers, Subway, Vasila)
18	Matero	Matero	Shoprite (Hungry Lion)
19	Novara Great North	Chazanga, SOS	PicknPay (GoatnChips, Hungry Lion)
20	PHI	Kaunda Square, PHI, Mtendere	PicknPay (Debonairs Pizza, King-Pie)
21	SOS Spurs	Chazanga, SOS	Spur
22	Twin Palm	Avondole, Chelston, Ibex, Salama Park	Shoprite (Chicken Inn, Debonairs Pizza, Hungry Lion)
23	Waterfalls	Avondole, Chelston	Shoprite (Gigibonta, Hungry Lion)
24	Woodlands	Chilenje, Kabulonga, Woodlands	PicknPay (Creamy, Debonairs Pizza, Galito's, Nachies, O. Hagans, Pizza Inn)
25	Zappa	Chawama	(Debonairs Pizza)

The main shopping malls that were operating in 2018 are included. Very small shopping malls were not included. Likewise, malls that were still under construction in 2018 were not included. The list was compiled by the authors based on internet search, personal visits, and key informant interviews.

Table S2. Distribution of sampled individuals in Lusaka City

Name of the Surveyed Compounds/Sections	Income Level	By Sex and Age Cohort				All
		Adult Females (≥18 years)	Adult Males (≥18 years)	Children/Adolescents (<18 years)		
				Girls	Boys	
Four: Avondole, Chalala, Kabulonga, and Woodlands	High	121	76	46	39	282
Four: Chelston, Chilenje, Kabwata, and PHI	Middle	250	122	94	93	559
Six: Chawama, Chazanga, Gardens, Kalingalinga, Kaunda Square, and Ng'ombe	Low	252	109	131	96	588
All		623	307	271	228	1429

Table S3. Food processing levels by food groups and items

Processing Level	Food Group	Food Items (Examples)
Unprocessed foods	Cereals and tubers	Maize (dry/green), cassava, irish potato, sweet potato, yams
	Eggs and milk	Eggs, fresh whole milk
	Fruits	Apples, avocado, banana (ripe/boiled), guava, mango, pawpaw, pineapple, pumpkin, orange/tangerine, sugar plum, watermelon
	Legumes	Bean (fresh/dry), cowpea (fresh/dry), groundnut (fresh/dry), pigeonpea (fresh/dry), soybean, velvet bean
	Vegetables	Bean leaves, blackjack, cabbage, carrot, cassava leaves, cowpea leaves, cucumber, eggplant, garlic, greengram, lettuce, mushroom (cultivated/wild), okra, onion, pepper, pumpkin leaves, rape/mustard/chinese, tomato
Primary processed foods	Drinks and snacks	Bottled/clear beer, bottled water, roasted cashew/macadamia nuts
	Meat and fish	Beef, bush/game meat, chicken, duck, turkey, goat meat, sheep meat, pork, fish (fresh/frozen/dried)
	Cereals	Rice, millet, oats, sorghum
Ultra-processed foods	Bread and pasta	Bread, buns, pasta, instant noodles
	Cereals and tubers	Maize flour, cornflakes, porridge mix, wheat flour, cassava flour
	Dairy products	Cheese, milk, yoghurt
	Oils and fats	Butter/margarine, coconut oil, cooking oil/fat
	Meat and fish	Sausage (beef/chicken/pork), soya meat, canned meat and fish
	Miscellaneous	Canned foods, mandazi, mixed fruits/salads, pizza, samosa, ready-made foods/dishes
	Sugar, sweetened drinks and snacks	Soft drinks, sweetened fruit juices, wine, jam, tomato sauce, salt, sugar, biscuits/cookies, cake, chips, chocolate, crisps, puffed salted corn chips, popcorn, salted nuts

Note: The same classifications of foods were also used by [10,11].

Table S4. Food groups and components used for construction of dietary quality indicators

Dietary Diversity Score			
**Group**	**Food Groups**	**Food Items (Examples)**	**Weight**
1	Cereals and tubers	Maize, rice, sorghum, millet, bread, cassava, potatoes, plantains	1
2	Pulses	Beans, cowpea, groundnuts, pigeonpea, soybean, velvet beans	1
3	Meat	Beef, chicken, ducks, goat meat, sheep meat, and pork	1
4	Fish	Fish (fresh/frozen/dried/tinned)	1
5	Eggs	Eggs	1
6	Vitamin A rich vegetables	Cassava leaves, sweet potato leaves	1
7	Other vegetables	Tomatoes, cabbage etc.	1
8	Fruits	Fruits	1
9	Dairy	Milk, yoghurt, and other dairy products	1

Healthy Eating Index (HEI)			
**Component**	**Standard for Maximum Score**	**Standard for Minimum Score (Zero)**	**Maximum Points**
Adequacy			
Total fruits	≥0·8 cup equivalent per 1000 kcal	No fruit	5
Whole fruits	≥0·4 cup equivalent per 1000 kcal	No whole fruit	5
Total vegetables	≥1·1 cup equivalent per 1000 kcal	No vegetables	5
Greens and beans	≥0·2 cup equivalent per 1000 kcal	No dark-green vegetables or pulses	5
Whole grains	≥1·5 ounce equivalent per 1000 kcal	No whole grains	10
Dairy	≥1·3 cup equivalent per 1000 kcal	No dairy	10
Total protein foods	≥2·5 ounce equivalent per 1000 kcal	No protein foods	5
Seafood and plant proteins	≥0·8 ounce equivalent per 1000 kcal	No seafood or plant proteins	5
Fatty acids [a]	(PUFAs+MUFAs)/SFAs≥2·5	(PUFAs+MUFAs)/SFAs≤1·2	10
Moderation			
Refined grains	≤1·8 ounce equivalent per 1000 kcal	≥4·3 ounce equivalent per 1000 kcal	10
Sodium	≤1·1 grams per 1000 kcal	≥2·0 grams per 1000 kcal	10
Added sugars	≤6·5% of energy	≥26% of energy	10
Saturated fats	≤8% of energy	≥16% of energy	10

[a] Ratio of poly-and mono-unsaturated fatty acids (PUFAs and MUFAs) to saturated fatty acids (SFAs). HEI components and weights based on [12–14].

Table S5. Estimated average requirements of calories and nutrients by sex and age cohort

Sex	Age (years)	Calorie and Nutrients [a]				
		**Calorie** [b]	**Protein** [d]	**Iron**	**Zinc** [e]	**Vitamin A**
		(kcal/day)	(g/day)	(mg/day)	(mg/day)	(μg retinol/day)
Child	1–3	531 [c]	10·0	5·8	2·0	200
	4–6	595 [c]	14·8	6·3	3·0	200
	7–9	680 [c]	21·0	8·9	5·0	250
	10–18	2200	36·4	8·0	8·6	330
Males	19–65	2400	55·5	11·0	10·0	300
	≥66	2200	54·6	8·0	10·0	300
	10–18	1600	38·6	8·0	7·0	330
Females	19–65 non-lactating	2000	54·9	18·0	6·0	270
	≥66	1800	57·1	8·0	6·0	300

[a] Estimated average requirements for nutrients are based on FAO [15], WHO and FAO [16], and IOM [17]. [b] Estimated average energy (calories) requirements for adults are based on IOM [18], assuming a moderate active individual. [c] Daily energy requirements obtained by averaging values for respective age groups from Table 3·2 in FAO [18]. [d] Anthropometric data (weight in kg) and the reference body weight of 0·8 g/kg/day [18] used as conversion factor to calculate daily protein requirements for individuals. [e] Zinc requirements are based in International Zinc Nutrition Consultative Group (IZiNCG) for mixed or refined vegetarian diets [19].

Table S6. Additional descriptive statistics for users and non-users of modern retailers

Variables	Units	Adults (≥18 years)		Children (<18 years)	
		Modern Retailers		Modern Retailers	
		Users	Non-users	Users	Non-users
		(N = 713)	(N = 217)	(N = 358)	(N = 141)
Dependent variables					
Mean adequacy ratio	Percent, %	91·51***	86·99	93·44	92·22
		(17·68)	(21·51)	(16·03)	(17·92)
Iron	Percent, %	171·96	175·87	227·98	245·23
		(103·61)	(101·25)	(122·89)	(157·20)
Zinc	Percent, %	108·69	108·93	116·23	119·10
		(85·02)	(84·95)	(77·49)	(136·35)
Vitamin A	Percent, %	185·79***	146·75	185·80*	150·76
		(176·86)	(165·23)	(202·58)	(176·71)
Treatment variable					
Modern retailer use	Expenditure share,%	59·30***	0·00	51·59***	0·00
		(31·13)	(0·00)	(30·49)	(0·00)
Control variables					
Household income	US$/capita/year	3265·18***	1041·53	2575·76***	866·82
		(3306·72)	(1212·42)	(3454·14)	(1141·88)
Male	1= Yes, 0 otherwise	0·33	0·33	0·46	0·45
		(0·47)	(0·47)	(0·50)	(0·50)
Age of household respondent	Years	34·84	36·56	8·98	8·59
		(13·97)	(15·08)	(4·87)	(4·73)
Education of respondent	Schooling years	12·27***	9·32	4·07**	3·29
		(3·18)	(3·39)	(3·93)	(3·39)
Household size	Number of members	4·47***	4·89	4·93	5·16
		(1·60)	(1·89)	(1·45)	(1·60)
Bemba as ethnicity	1= Yes, 0 otherwise	0·29**	0·20	0·34*	0·26
		(0·45)	(0·40)	(0·47)	(0·44)
Chewa as ethnicity	1= Yes, 0 otherwise	0·12**	0·18	0·12**	0·18
		(0·33)	(0·38)	(0·32)	(0·39)
Tonga as ethnicity	1= Yes, 0 otherwise	0·21	0·20	0·15	0·17
		(0·41)	(0·40)	(0·35)	(0·38)
Protestant as a religion	1= Yes, 0 otherwise	0·44	0·41	0·49	0·45
		(0·50)	(0·49)	(0·50)	(0·50)
Catholic as a religion	1= Yes, 0 otherwise	0·25**	0·32	0·18***	0·33
		(0·43)	(0·47)	(0·39)	(0·47)
Physical activity ratio	Ratio; range (0·03–15·95)	2·74	2·76	3·52	3·58
		(1·45)	(1·71)	(2·26)	(2·35)
Piped or tap drinking water	1= Yes, 0 otherwise	0·94**	0·88	0·92*	0·87
		(0·24)	(0·32)	(0·27)	(0·34)
Non-chronic infections	1= Yes, 0 otherwise	0·27	0·26	0·27*	0·19
		(0·44)	(0·44)	(0·45)	(0·39)
Distance to the nearest	km; range (0·01–30)	2·38***	1·90	2·32	2.07
		(2·06)	(1·81)	(2·01)	(3·00)
High quality food products	1= Yes, 0 otherwise	0·23***	0·01	0·18***	0·04
		(0·42)	(0·12)	(0·38)	(0·19)
Neighbor's shopping mall	Number of visits per week	2·88***	1·68	3·00***	1·48
		(2·91)	(2·25)	(3·04)	(2·31)
Household distance to a shopping mall	GPS-measured distance in km	2·57***	3·59	2·53***	3·88
		(2·57)	(2·26)	(2·50)	(2·49)

Mean values are shown with standard deviations in parentheses. The average exchange rate was ZMW 9·87 = US$ 1 in mid-2018. *t*-tests were used to test for mean differences between users and non-users of modern retailers. * p < 0·10, ** p < 0·05, *** p < 0·01.

Table S7. Household expenditure shares for foods at different processing levels

	Overall	Modern Retailers		Mean Difference
		Users	Non-users	
	(1)	(2)	(3)	(4) = (2) – (3)
Unprocessed foods (%)	24·70	22·97	30·14	–7·17***
	(14·17)	(12·77)	(16·80)	(1·48)
Primary processed foods (%)	40·10	41·96	34·28	7·68***
	(17·13)	(16·26)	(18·48)	(1·80)
Ultra-processed foods (%)	35·19	35·07	35·58	–0·51
	(13·52)	(12·97)	(15·16)	(1·45)
N	475	360	115	475

Mean values are shown with standard deviations in parentheses (standard errors in column 4). *** $p < 0{\cdot}01$. N, number of observations.

Table S8. Effects of using modern retailers on dietary composition in terms of food processing levels (full model results for Table 3)

	**Unprocessed Foods** (expenditure share, %)		**Primary Processed Foods** (expenditure share, %)		**Ultra-Processed Foods** (expenditure share, %)	
	OLS	CF	OLS	CF	OLS	CF
	(1)	(2)	(3)	(4)	(5)	(6)
Modern retail use	–0·071**	–0·060	0·018	–0·005	0·053*	0·065
(expenditure share, %)	(0·027)	(0·058)	(0·023)	(0·079)	(0·025)	(0·063)
Male household head (dummy)	0·755	0·870	–0·619	–0·866	–0·136	–0·004
	(1·899)	(1·317)	(1·934)	(1·758)	(1·402)	(1·337)
Age of household head (HH) (years)	–0·425	–0·410	0·684*	0·653*	–0·259	–0·242
	(0·255)	(0·264)	(0·359)	(0·358)	(0·364)	(0·305)
Age of HH-squared (years)	0·005*	0·005*	–0·008*	–0·008**	0·003	0·003
	(0·003)	(0·003)	(0·004)	(0·004)	(0·004)	(0·003)
Education of HH (years)	–0·140	–0·179	0·726***	0·808**	–0·585***	–0·630**
	(0·184)	(0·289)	(0·214)	(0·371)	(0·120)	(0·302)
Household size (individuals)	0·385	0·399	–1·159**	–1·189**	0·774**	0·790*
	(0·386)	(0·422)	(0·417)	(0·539)	(0·349)	(0·439)
Household income (log)	–1·680**	–1·794*	1·618*	1·864	0·062	–0·070
	(0·708)	(0·924)	(0·814)	(1·218)	(0·490)	(1·042)
Chewa (dummy)	–2·766	–2·803	2·575	2·654	0·191	0·149
	(2·520)	(2·114)	(2·038)	(2·223)	(1·630)	(1·889)
Tonga (dummy)	–0·623	–0·593	1·078	1·013	–0·455	–0·420
	(1·524)	(1·415)	(1·996)	(2·078)	(1·480)	(1·831)
Catholic (dummy)	0·728	0·755	–0·105	–0·162	–0·623	–0·592
	(1·108)	(1·415)	(1·808)	(1·827)	(2·155)	(1·256)
First-stage residual		–0·012		0·026		–0·014
		(0·062)		(0·085)		(0·067)
Constant	51·226***	51·826***	6·935	5·642	41·839***	42·532***
	(7·804)	(9·299)	(8·830)	(10·657)	(10·118)	(9·401)
Joint F-statistic/Wald χ^2	9***	72***	12***	81***	5**	18*
R^2	0·135	0·135	0·127	0·127	0·033	0·033
N	475	475	475	475	475	475

OLS (ordinary least squares) and control function (CF) estimates are shown with robust and bootstrapped standard errors clustered at compound level in parentheses, respectively. Bemba and Protestant are used as reference group for ethnicity—Chewa and Tonga, and religion status—Catholic, respectively. * p < 0·10, ** p < 0·05, *** p < 0·01. N, number of observations (households).

Table S9. Effects of modern retailers on dietary composition in terms of food processing levels (alternative model specifications)

	**Unprocessed Foods** (expenditure share, %)	**Primary Processed Foods** (expenditure share, %)	**Ultra-Processed Foods** (expenditure share, %)
	(1)	(2)	(3)
Estimated with Heckman selection model			
Modern retail use	–0·065***	0·008	0·079***
(expenditure share, %)	(0·024)	(0·033)	(0·027)
Control variables	Yes	Yes	Yes
N	475	475	475
Estimated with OLS, only including modern retail users			
Modern retail use	–0·090**	0·010	0·081**
(expenditure share, %)	(0·035)	(0·030)	(0·033)
Control variables	Yes	Yes	Yes
N	360	360	360
Estimated with OLS, expressing modern retailer use as a dummy			
Modern retail use	–2·982*	2·319	0·663
(dummy)	(1·630)	(2·133)	(2·554)
Control variables	Yes	Yes	Yes
N	475	475	475

Marginal effects are shown with robust standard errors clustered at compound level in parentheses. In all models, the same control variables as shown in Supplementary Table S8 were used in estimation. ** $p < 0·05$, *** $p < 0·01$. N, number of household observations.

Table S10. Effects of modern retailers on nutritional status (full model results for Table 4)

	Adults (≥18 years)		Children (<18 years)			Adults (≥18 years)		Children (<18 years)		
	**BMI**	**Overweight /Obese**	**BAZ**	**Overweight /Obese**	**HAZ**	**BMI**	**Overweight /Obese**	**BAZ**	**Overweight /Obese**	**HAZ**
	OLS	Probit	OLS	Probit	OLS	CF	CF	CF	CF	CF
	(1)	(2)	(3)	(4)	(5)	(6)	(7)	(8)	(9)	(10)
Modern retail use	0·012**	0·004***	0·001	–0·002	0·006**	0·004	0·003	–0·011	–0·016**	0·026***
(expenditure share, %)	(0·005)	–0.002	(0·002)	(0·004)	(0·003)	(0·014)	(0·005)	(0·008)	(0·007)	(0·008)
Male (dummy)	–0·900**	–0·284***	0·558**	0·410*	–0.083	–0·930***	–0·290**	0·566***	0·440**	–0·097
	(0·349)	(0·104)	(0·217)	(0·228)	(0·186)	(0·341)	(0·117)	(0·190)	(0·179)	(0·206)
Age (years)	0·374***	0·101***	–0·154**	–0·144	0·273***	0·371***	0·101***	–0·132*	–0·116	0·236***
	(0·062)	(0·022)	(0·067)	(0·094)	(0·063)	(0·054)	(0·016)	(0·074)	(0·076)	(0·077)
Age-squared (years)	–0·003***	–0·001***	0·003	0·003	–0·022***	–0·003***	–0·001***	0·002	0·002	–0·020***
	(0·001)	(0·0002)	(0·003)	(0·004)	(0·002)	(0·001)	(0·000)	(0·004)	(0·004)	(0·004)
Education (years)	–0·099	–0·0003	0·127**	0·111**	0·114**	–0·077	0·004	0·130***	0·113*	0·109**
	(0·080)	(0·017)	(0·045)	(0·050)	(0·053)	(0·070)	(0·023)	(0·040)	(0·060)	(0·049)
Household size (individuals)	0.086	0·020	–0·022	–0·036	0·005	0·071	0·017	–0·038	–0·058	0·029
	(0·073)	(0·030)	(0·041)	(0·049)	(0·047)	(0·104)	(0·029)	(0·047)	(0·052)	(0·047)
Income (log)	0·079	0·039	0·025	0·064	–0·055	0·172	0·056	0·205	0·283**	–0·365***
	(0·206)	(0·056)	(0·112)	(0·094)	(0·066)	(0·247)	(0·077)	(0·146)	(0·127)	(0·140)
Chewa (dummy)	0·061	0·278	–0·220	–0·315*	–0·085	0·061	0·278**	–0·263	–0·396	–0·006
	(0·555)	(0·183)	(0·182)	(0·165)	(0·136)	(0·412)	(0·134)	(0·214)	(0·250)	(0·148)
Tonga (dummy)	0·373	0·145	0·088	–0·151	0·014	0·334	0·137	0·092	–0·152	0·015
	(0·449)	(0·106)	(0·193)	(0·211)	(0·236)	(0·404)	(0·119)	(0·217)	(0·203)	(0·249)
Catholic (dummy)	0·387	0·025	0·018	0·018	–0·105	0·367	0·022	–0·061	–0·068	0·036
	(0·275)	(0·113)	(0·174)	(0·200)	(0·170)	(0·301)	(0·110)	(0·158)	(0·197)	(0·146)
Physical activity ratio	–0·843***	–0·215***	–0·140***	–0·111**	–0·086*	–0·840***	–0·215***	–0·132***	–0·107**	–0·098**
	(0·120)	(0·031)	(0·038)	(0·052)	(0·048)	(0·116)	(0·039)	(0·036)	(0·043)	(0·041)
Piped drinking water					0·263					0·214
					(0·335)					(0·222)
Non-chronic infections					–0·239					–0·182
					(0·139)					(0·149)
Distance to the nearest hospital					–0·039*					–0·038*
(km)					(0·021)					(0·022)
Constant	19·016***	–2·420***	0·628	–0·549	–0·631	18·353***	–2·548***	–0·613	–2·049**	1·527
	(2·341)	(0·595)	(1·068)	(1·096)	(0·807)	(2·360)	(0·756)	(1·223)	(0·997)	(1·062)
First-stage residual						0·008	0·002	0·013*	0·016**	–0·023***
						(0·015)	(0·005)	(0·007)	(0·007)	(0·008)
Joint F-statistic/Wald χ^2	761***	862***	37***	192***		327***	162***	66***	35***	117***
R^2/Pseudo-R^2	0·220	0·146	0·057	0·046	0·133	0·220	0·147	0·063	0·055	0·151
N	863	863	458	458	472	863	863	458	458	472

Coefficient estimates are shown with robust, bootstrapped standard errors clustered at compound level in parentheses. OLS estimator. Bemba and Protestant are used as reference group for ethnicity—Chewa and Tonga, and religion status—Catholic, respectively. * p < 0·10, ** p < 0·05, *** p < 0·01.

Table S11. Effects of modern retailers on nutritional status (Heckman selection model results)

	Adults (≥18 years)		Children (<18 years)		
	**BMI**	**Overweight/ Obese**	**BAZ**	**Overweight/ Obese**	**HAZ**
	(1)	(2)	(3)	(4)	(5)
Modern retail use	0·014**	0·002**	–0·001	–0·001	0·007**
(expenditure share, %)	(0·006)	(0·001)	(0·003)	(0·056)	(0·003)
Control variables	Yes	Yes	Yes	Yes	Yes
N	872	872	470	470	479

Coefficient estimates are shown with robust standard errors in parentheses. In all models, the same control variables as shown in Supplementary Table S10 were used in estimation. ** $p < 0{\cdot}05$.

Table S12. Effects of modern retailers on adult dietary diversity (full model results for Figure 2)

	**HEI** Score (0–100) Poisson	**FVS** Score (0–18) Poisson	**DDS** Score (0–9) Poisson	**HEI** Score (0–100) CF	**FVS** Score (0–18) CF	**DDS** Score (0–9) CF
	(1)	(2)	(3)	(4)	(5)	(6)
Modern retail use	1·109***	1·038	1·040	1·193***	1·102**	1·118**
(dummy)	(0·039)	(0·026)	(0·041)	(0·041)	(0·039)	(0·039)
Male	–0·994	–0·995	1·019	1·001	1·001	1·025
	(0·027)	(0·021)	(0·027)	(0·022)	(0·020)	(0·024)
Age	–0·999	1·001	–0·999	–0·999	1·001	–0·999
	(0·004)	(0·003)	(0·003)	(0.004)	(0·003)	(0·004)
Age-squared	1·000	–1.000	1·000	–1·000	–1·000	1·000
	(0·00005)	(0·003)	(0·00004)	(0·00004)	(0·00003)	(0·00004)
Education	–0·990*	–0·999	–0·999	–0·988**	–0·997	–0·997
	(0·005)	(0·004)	(0·005)	(0·005)	(0·003)	(0·003)
Household size	–0·994	1·004	1·008	–0·993	1·003	1·007
	(0·008)	(0·008)	(0·006)	(0·008)	(0·007)	(0·007)
Income	1·012	1·023*	1·007	1·003	1·016	–0·998
	(0·016)	(0·013)	(0·013)	(0·011)	(0·010)	(0·012)
Chewa	1·027	1·049	1·057	1·029	1·050	1·059
	(0·036)	(0·031)	(0·039)	(0·033)	(0·034)	(0·038)
Tonga	1·078**	1·049	1·024	1·084***	1·054**	1·030
	(0·037)	(0·033)	(0·036)	(0·026)	(0·023)	(0·024)
Catholic	1·053	1·027	1·067**	1·056**	1·030	1·070***
	(0·034)	(0·026)	(0·021)	(0·028)	(0·026)	(0·024)
Constant	30·469***	4·845***	2·777***	32·133***	5·061***	2·931***
	(0·034)	(0·699)	(0·354)	(4·518)	(0·543)	(0·391)
First-stage residual				–0·998***	–0·998***	–0·998***
				(0·0004)	(0·0004)	(0·001)
Wald χ^2	123***	130***	56***	56***	41***	43***
Pseudo-R^2	0·013	0·003	0·002	0·021	0·005	0·004
N	930	930	930	930	930	930

Incidence rate ratios are shown with bootstrapped standard errors clustered at compound level in parentheses. For Figure 2, incidence ratios and standard errors were converted for percentage interpretation. CF, control function approach; HEI, healthy eating index; FVS, food variety score; DDS, dietary diversity score. Bemba and Protestant are used as a reference group for ethnicity—Chewa and Tonga, and religion status—Catholic, respectively. * $p < 0·10$, ** $p < 0·05$, *** $p < 0·01$.

Table S13. Effects of modern retailers on child dietary diversity (full model results for Figure 2)

	HEI Score (0–100) Poisson	FVS Score (0–18) Poisson	DDS Score (0–9) Poisson	HEI Score (0–100) CF	FVS Score (0–18) CF	DDS Score (0–9) CF
	(1)	(2)	(3)	(4)	(5)	(6)
Modern retail use	1·109***	1·049	–0·974	1·165***	1·049	1·014
(dummy)	(0·033)	(0·036)	(0·039)	(0·053)	(0·034)	(0·042)
Male	1·075***	–0·998	1·054*	1·075**	–0·998	1·054
	(0·025)	(0·019)	(0·031)	(0·033)	(0·025)	(0·034)
Age	1·061***	1·025**	1·032**	1·061***	1·025*	1·031
	(0·021)	(0·010)	(0·014)	(0·018)	(0·015)	(0·017)
Age-squared	–0·998*	–0·999	–0·999	–0·998**	–0·999	–0·999
	(0·001)	(0·001)	(0·001)	(0·001)	(0·001)	(0·001)
Education	–0·997	–0·983	–0·980	–0·996	–0·983	–0·979*
	(0·018)	(0·015)	(0·014)	(0·012)	(0·014)	(0·011)
Household size	–0·999	1·021***	1·020**	–0·999	1·021**	1·020**
	(0·010)	(0·007)	(0·009)	(0·012)	(0·009)	(0·010)
Income	1·017	1·040**	1·030	1·010	1·040***	1·023**
	(0·020)	(0·018)	(0·021)	(0·013)	(0·011)	(0·012)
Chewa	1·118**	1·097*	1·162***	1·122**	1·097***	1·165***
	(0·060)	(0·052)	(0·062)	(0·051)	(0·034)	(0·054)
Tonga	1·086	1·095***	1·068**	1·092*	1·095***	1·072*
	(0·065)	(0·034)	(0·031)	(0·049)	(0·037)	(0·043)
Catholic	1·055**	1·053**	1·019	1·063*	1·053**	1·025
	(0·027)	(0·026)	(0·032)	(0·035)	(0·025)	(0·031)
Constant	17·303***	3·495***	1·745***	17·861***	3·493***	1·794***
	(3·180)	(0·510)	(0·339)	(2·673)	(0·440)	(0·241)
First-stage residual				–0·999**	1·000	–0·999*
				(0·001)	(0·001)	(0·001)
Wald χ^2	216***	47***	259***	53***	57***	42***
Pseudo-R^2	0·040	0·009	0·007	0·043	0·009	0·008
N	499	499	499	499	499	499

Incidence rate ratios are shown with bootstrapped standard errors clustered at compound level in parentheses. For Figure 2, incidence ratios and standard errors were converted for percentage interpretation. CF, control function approach; HEI, healthy eating index; FVS, food variety score; DDS, dietary diversity score. Bemba and Protestant are used as a reference group for ethnicity—Chewa and Tonga, and religion status—Catholic, respectively. * p < 0·10, ** p < 0·05, *** p < 0·01.

Table S14. Effects of modern retailers on adult calorie and nutrient intakes (full model results for Figure 3)

	**Calorie** (kcal/day) Tobit	**Protein** (g/day) Tobit	**Iron** (mg/day) Tobit	**Zinc** (mg/day) Tobit	**Vitamin A** (μg/day) Tobit	**Calorie** (kcal/day) CF	**Protein** (g/day) CF	**Iron** (mg/day) CF	**Zinc** (mg/day) CF	**Vitamin A** (μg/day) CF
	(1)	(2)	(3)	(4)	(5)	(6)	(7)	(8)	(9)	(10)
Modern retail use	3·556***	0·096**	0·028**	0·008**	0·697*	13·286***	0·604***	0·164***	0·037***	1·267*
(expenditure share, %)	(0·916)	(0.046)	(0·013)	(0·004)	(0·409)	(3·517)	(0·099)	(0·037)	(0·010)	(0·739)
Male	269·499***	6·759***	1·466***	0·666***	–14·677	306·203***	8·705***	2·073***	0·773***	9·006
	(71·397)	(1·470)	(0·331)	(0·224)	(26·302)	(77·809)	(2·183)	(0·674)	(0·223)	(13·645)
Age	21·424*	0·639	0·029	0·085**	–0·275	25·303***	0.848**	0·088	0·096***	1·795
	(12·323)	(0·409)	(0·091)	(0·034)	(3·036)	(9·497)	(0·382)	(0·092)	(0·032)	(2·243)
Age-squared	–0·305**	–0·008*	–0·001	–0·001***	–0·009	–0·347***	–0·010**	–0·001	–0·001***	–0·024
	(0·132)	(0·005)	(0·001)	(0·0004)	(0·036)	(0·106)	(0·005)	(0·001)	(0·0004)	(0·027)
Education	–22·152***	–0·755***	–0·087	–0·059*	4·750	–50·816***	–2·256***	–0·480***	–0·144***	–0·308
	(8·361)	(0·244)	(0·074)	(0·033)	(2·966)	(17·861)	(0·547)	(0·154)	(0·053)	(3·107)
Household size	–40·738	–3·180**	–0·388	–0·253**	–14·600**	–19·778	–2·080***	–0·100	–0·192***	–7·428*
	(36·400)	(1·305)	(0·250)	(0·117)	(5·918)	(22·973)	(0·739)	(0·202)	(0·072)	(3·896)
Income	45·815	1·182	0·205	0·040	22·066	–76·970	–5·222***	–1·514***	–0·322**	1·923
	(37·039)	(1·035)	(0·356)	(0·106)	(13·680)	(52·564)	(1·522)	(0·542)	(0·158)	(10·781)
Chewa	–178·209*	0·603	0·044	–0·364	–7·026	–180·927*	0·425	–0·063	–0·370	9·029
	(95·287)	(4·727)	(1·063)	(0·434)	(21·073)	(100·034)	(3·692)	(1·029)	(0·339)	(20·210)
Tonga	162·359**	10·934***	2·943***	0·540**	4·938	212·855**	13·568***	3·739***	0·688**	23·457
	(81·023)	(2·299)	(0·773)	(0·269)	(26·619)	(96·257)	(2·922)	(0·938)	(0·325)	(17·390)
Catholic	109·489	7·143**	1·091	0·593**	72·108*	134·599*	8·469***	1·466**	0·667**	41·942***
	(71·476)	(2·925)	(0·751)	(0·246)	(37·249)	(81·645)	(2·691)	(0·747)	(0·263)	(14·340)
Constant	1990·091***	70·085***	17·915***	6·350***	164·583	2854·905***	115·080***	29·946***	8·902***	190·720**
	(602·489)	(16·065)	(3·279)	(1·622)	(149·339)	(501·210)	(15·024)	(4·638)	(1·639)	(94·957)
First-stage residual						–11·004***	–0·574***	–0·150***	–0·033***	–0·637
						(3·836)	(0·108)	(0·039)	(0·011)	(0·787)
Joint F-statistic	11***	202***	43***	8***	12***					
Wald χ^2						103***	199***	78***	98***	88***
Pseudo-R^2	0·004	0·010	0·024	0·011	0·006	0·004	0·013	0·033	0·012	0·009
N	930	930	930	930	930	930	930	930	930	930

Coefficient estimates are shown with bootstrapped standard errors clustered at compound level in parentheses. Estimates for modern retail use can be interpreted as marginal effects of a 1 percentage point increase in the modern retail expenditure share. For Figure 3, coefficients and standard errors were multiplied by 10, to show effects of a 10 percentage point increase in the modern retail expenditure share. CF, control function approach. Bemba and Protestant are used as reference group for ethnicity—Chewa and Tonga, and religion status—Catholic, respectively. * $p < 0·10$, ** $p < 0·05$, *** $p < 0·01$.

Table S15. Effects of modern retailers on child calorie and nutrient intakes (full model results for Figure 3)

	**Calorie** (kcal/day) Tobit	**Protein** (g/day) Tobit	**Iron** (mg/day) Tobit	**Zinc** (mg/day) Tobit	**Vitamin A** (µg/day) Tobit	**Calorie** (kcal/day) CF	**Protein** (g/day) CF	**Iron** (mg/day) CF	**Zinc** (mg/day) CF	**Vitamin A** (µg/day) CF
	(1)	(2)	(3)	(4)	(5)	(6)	(7)	(8)	(9)	(10)
Modern retail use	2·455*	0·018	0·021**	0·010**	0·405	9·662**	0·346***	0·114***	0·027**	1·293*
(expenditure share, %)	(1.413)	(0·061)	(0·009)	(0·004)	(0·653)	(3·865)	(0·128)	(0·035)	(0·014)	(0·756)
Male	139·026**	5·023	1.032**	0·315	12·135	107·787	4·059	1·445**	0·270	–0·908
	(69·052)	(3·727)	(0·485)	(0·317)	(20·830)	(67·943)	(2·474)	(0·628)	(0·224)	(16·540)
Age	197·369***	6·622***	2·087***	0·660***	–10·283	166·034***	5·965***	1·756***	0·626***	–10·561
	(23·568)	(1·047)	(0·414)	(0·130)	(9·907)	(36·470)	(1·233)	(0·332)	(0·131)	(8·765)
Age-squared	–7·327***	–0·198***	–0·082***	–0·019**	0·413	–5·845***	–0·162***	–0·057***	–0·017**	0·370
	(1·236)	(0·064)	(0·015)	(0·007)	(0·516)	(2·030)	(0·063)	(0·019)	(0·007)	(0·445)
Education	8·979	–0·336	–0·239	–0·049	6·473	4·290	–0·423	–0·100	–0·052	6·454
	(34·658)	(0·779)	(0·372)	(0·044)	(10·394)	(33·179)	(0·936)	(0·276)	(0·084)	(5·801)
Household size	3·084	–0·783	–0·080	–0·138	–26·300***	13·519	–0·358	–0·141	–0·117	–11·612**
	(27·250)	(1·087)	(0·248)	(0·124)	(7·226)	(24·648)	(0·701)	(0·222)	(0·073)	(4·932)
Income	38·802	0·856	0·035	0·083	59·428***	–76·856	–4·171**	–1·348**	–0·180	16·124
	(62·287)	(0·965)	(0·411)	(0·096)	(13·054)	(65·738)	(2·078)	(0·557)	(0·245)	(14·000)
Chewa	86·984	8·839	–1·169	0·313	41·493	111·387	10·019**	1·307	0·368	41·133
	(223·630)	(5·999)	(1·227)	(0·427)	(50·216)	(112·804)	(4·131)	(1·020)	(0·413)	(26·894)
Tonga	–52·352	6·526	0·006	0·243	1·015	–37·812	6·698*	0·974	0·245	23·155
	(113·153)	(5·805)	(0·530)	(0·386)	(44·334)	(106·656)	(3·495)	(0·922)	(0·370)	(17·906)
Catholic	95·974	3·274	0·657	0·445	42·909	124·233	5.349*	2·245**	0·554*	29·816
	(126·386)	(3·647)	(0·966)	(0·393)	(36·907)	(98·235)	(3·084)	(0·945)	(0·323)	(21·011)
Constant	272·873	12·095	2·892	0·454	–84·023	1124·865**	46·557***	13·317***	2.259	135·035
	(518·702)	(8·614)	(3·629)	(1·081)	(117·647)	(522·421)	(16·289)	(4·423)	(2.057)	(117·268)
First-stage residual						–8·618**	–0·371***	–0·113***	–0·019	–1·105
						(4·191)	(0·130)	(0·037)	(0·014)	(0·866)
Joint F-statistic	37***	108***	29***	64***	10***					
Wald χ^2						130***	203***	163***	194***	89***
Pseudo-R^2	0·044	0·030	0·134	0·057	0·011	0·041	0·031	0·081	0·058	0·013
N	499	499	499	499	499	499	499	499	499	499

Coefficient estimates are shown with bootstrapped standard errors clustered at compound level in parentheses. Estimates for modern retail use can be interpreted as marginal effects of a 1 percentage point increase in the modern retail expenditure share. For Figure 3, coefficients and standard errors were multiplied by 10, to show effects of a 10 percentage point increase in the modern retail expenditure share. CF, control function approach. Bemba and Protestant are used as reference group for ethnicity—Chewa and Tonga, and religion status—Catholic, respectively. * p < 0·10, ** p < 0·05, *** p < 0·01.

Table S16. Effects of modern retailers on adult micronutrient adequacy ratios

	Mean Adequacy Ratio	Micronutrient Adequacy Ratio		
		Iron	Zinc	Vitamin A
	(1)	(2)	(3)	(4)
Modern retail use	0·245***	1·073***	0·386*	–0·033
(expenditure share, %)	(0·046)	(0·283)	(0·230)	(0·660)
Male	3·739***	91·887***	–40·047***	–23·791*
	(1·117)	(7·163)	(5·657)	(12·423)
Age	–0·344**	–4·543***	1·120	1·057
	(0·171)	(1·150)	(0·789)	(1·988)
Age-squared	0·003*	0·060***	–0·016*	–0·020
	(0·002)	(0·014)	(0·008)	(0·023)
Education	–0·563*	–3·450**	–1·553	3·983
	(0·288)	(1·431)	(1·199)	(2·642)
Household size	–0·545	–3·071*	–4·171***	–6·718*
	(0·465)	(1·752)	(1·339)	(3·503)
Income	–0·688	–10·273***	–5·625*	15·838
	(0·859)	(3·761)	(3·340)	(10·274)
Chewa	–0·827	–3·285	–5·520	–27·278*
	(2·194)	(8·198)	(7·358)	(14·103)
Tonga	3·019**	32·493***	7·424	–2·784
	(1·237)	(8·499)	(8·037)	(16·177)
Catholic	2·190	16·228***	11·188*	34·900***
	(1·389)	(5·854)	(5·967)	(12·633)
Constant	99·622***	309·095***	175·356***	4·742
	(8·760)	(38·222)	(34·081)	(90·443)
First-stage residual	–0·210***	–0·997***	–0·321	0·210
	(0·050)	(0·289)	(0·246)	(0·718)
Wald χ^2 (11)	142***	296***	121***	73***
R^2	0·093	0·233	0·070	0·053
N	930	930	930	930

Coefficient estimates from control function models are shown with bootstrapped standard errors clustered at compound level in parentheses. * p < 0·10, ** p < 0·05, *** p < 0·01.

Table S17. Effects of modern retailers on child micronutrient adequacy ratios

		Micronutrient Adequacy Ratio		
	**Mean Adequacy Ratio**	**Iron**	**Zinc**	**Vitamin A**
	(1)	(2)	(3)	(4)
Modern retail use	0·179***	1·380**	0·849**	0·072
(expenditure share, %)	(0·069)	(0·659)	(0·428)	(0·945)
Male	2·823**	25·477**	0·283	11·346
	(1·408)	(12·476)	(7·283)	(17·115)
Age	0·800	10·011*	–11·086***	–17·490*
	(0·997)	(5·237)	(3·748)	(9·156)
Age-squared	–0·028	–0·113	0·473***	0·606
	(0·043)	(0·283)	(0·168)	(0·460)
Education	–0·082	–1·419	–4·091*	–1·268
	(0·482)	(3·613)	(2·323)	(4·630)
Household size	–0·383	–5·487*	–2·659	–11·848**
	(0·555)	(3·190)	(2·119)	(5·390)
Income	–0·476	–21·234**	–10·676	41·194**
	(1·316)	(10·194)	(8·768)	(16·821)
Chewa	–0·145	31·016	10·337	39·890
	(2·119)	(20·533)	(11·368)	(30·429)
Tonga	1·032	43·547**	20·892	–8·811
	(1·669)	(18·522)	(18·827)	(16·828)
Catholic	2·497	36·843***	13·791	5·847
	(1·837)	(14·034)	(9·011)	(20·482)
Constant	86·888***	303·276***	255·781***	–61·510
	(11·732)	(79·592)	(76·754)	(137·665)
First-stage residual	–0·165**	–1·411**	–0·676	0·420
	(0·075)	(0·682)	(0·482)	(1·019)
Wald χ^2 (11)	38***	92***	69***	78***
R^2	0·067	0·117	0·097	0·132
N	499	499	499	499

Coefficient estimates from control function models are shown with bootstrapped standard errors clustered at compound level in parentheses. * p < 0·10, ** p < 0·05, *** p < 0·01.

Table S18. Effects of supermarkets on child and adult calorie and nutrient intakes

	**Calorie** (kcal/day)	**Protein** (g/day)	**Iron** (mg/day)	**Zinc** (mg/day)	**Vitamin A** (µg retinol/day)
	(1)	(2)	(3)	(4)	(5)
**Adults**					
Supermarket use	10·430***	0·497***	0·139***	0·029***	1·029**
(expenditure share, %)	(2·882)	(0·094)	(0·031)	(0·009)	(0·488)
Controls	Yes	Yes	Yes	Yes	Yes
First-stage residual	–7·724**	–0·440***	–0·118***	–0·024**	–0·424
	(3·237)	(0·103)	(0·034)	(0·009)	(0·525)
Wald χ^2 (11)	76***	133***	53***	72***	66***
Pseudo-R^2	0·004	0·013	0·034	0·012	0·009
N	930	930	930	930	930
**Children**					
Supermarket use	8·276**	0·295**	0·102***	0·022*	1·106
(expenditure share, %)	(3·595)	(0·119)	(0·029)	(0·013)	(0·722)
Controls	Yes	Yes	Yes	Yes	Yes
First-stage residual	–8·810**	–0·324***	–0·104***	–0·015	–0·843
	(3·700)	(0·123)	(0·033)	(0·013)	(0·781)
Wald χ^2 (11)	127***	220***	188***	217***	88***
Pseudo-R^2	0·041	0·031	0·081	0·057	0·013
N	499	499	499	499	499

Coefficient estimates from control function models are shown with bootstrapped standard errors clustered at compound level in parentheses. The same control variables as those shown in Supplementary Tables S14 and S15 were included in estimation. * p < 0·10, ** p < 0·05, *** p < 0·01.

Table S19. Effects of modern retailers on calorie and nutrient intakes of individuals from poor households (model results for Figure 4)

	**Calorie** (kcal/day)	**Protein** (g/day)	**Iron** (mg/day)	**Zinc** (mg/day)	**Vitamin A** (µg retinol/day)
	(1)	(2)	(3)	(4)	(5)
**Adults**					
Modern retail use	17·070**	0·757***	0·244***	0·045*	3·615**
(expenditure share, %)	(7·656)	(0·234)	(0·065)	(0·025)	(1·470)
Controls	Yes	Yes	Yes	Yes	Yes
First-stage residual	–18·272**	–0·760***	–0·260***	–0·059*	–2·541
	(8·971)	(0·277)	(0·073)	(0·034)	(1·668)
Wald χ^2 (11)	14	32***	24**	17***	51***
Pseudo-R^2	0·003	0·012	0·026	0·010	0·012
N	226	226	226	226	226
**Children**					
Modern retail use	22·962***	0·558***	0·118**	0·026	3·716***
(expenditure share, %)	(5·310)	(0·199)	(0·060)	(0·026)	(1·096)
Controls	Yes	Yes	Yes	Yes	Yes
First-stage residual	–22·306***	–0·653***	–0·147**	–0·032	–3·064**
	(5·857)	(0·207)	(0·065)	(0·030)	(1·250)
Wald χ^2 (11)	61***	164***	62***	76***	35***
Pseudo-R^2	0·058	0·039	0·103	0·057	0·016
N	175	175	175	175	175

Poor households are defined as those with incomes less than $1·90 per capita and day. Coefficient estimates from control function models are shown with bootstrapped standard errors clustered at compound level in parentheses. The same control variables as those shown in Supplementary Tables S14 and S15 were included in estimation. * p < 0·10, ** p < 0·05, *** p < 0·01.

Table S20. Effects of modern retailers on calorie and nutrient intakes disaggregated by sex

	**Calorie** (kcal/day)	**Protein** (g/day)	**Iron** (mg/day)	**Zinc** (mg/day)	**Vitamin A** (μg/day)
	(1)	(2)	(3)	(4)	(5)
Effects on adult males	11·802*	0·550***	0·221**	0·035*	1·556
	(6·963)	(0·201)	(0·106)	(0·019)	(0·972)
N	307	307	307	307	307
Effects on adult females	13·464***	0·628***	0·140***	0·036***	1·056
	(4·498)	(0·136)	(0·037)	(0·012)	(0·816)
N	623	623	623	623	623
Effects on boys	13·375**	0·238	0·103**	0·018	0·571
	(6·359)	(0·163)	(0·051)	(0·019)	(1·336)
N	228	228	228	228	228
Effects on girls	7·399	0·448**	0·138***	0·040*	1·964
	(6·419)	(0·218)	(0·052)	(0·023)	(1·278)
N	271	271	271	271	271

Coefficient estimates from control function models are shown with bootstrapped standard errors clustered at compound level in parentheses. The treatment variable in all models is the share of total food expenditures (in %) made in modern retailers. The same control variables as those shown in Supplementary Tables S14 and S15 were included in estimation. * $p < 0·10$, ** $p < 0·05$, *** $p < 0·01$.

Table S21. First-stage estimation results on food purchases in modern retailers

	**Modern Retail Use** (expenditure share, %)	
	**Adults**	**Children**
	(1)	(2)
Male (dummy)	–4·493**	3·666
	(1·958)	(2·465)
Age of respondent (years)	–0·430	2·284**
	(0·312)	(1·161)
Age-squared (years)	0·005	–0·095
	(0·004)	(0·065)
Education of respondent (years)	2·611***	–0·435
	(0·304)	(0·941)
Household size (individuals)	–1·870***	–0·953
	(0·561)	(0·840)
Income (log)	10·992***	12·821***
	(0·835)	(1·100)
Chewa (dummy)	1·347	–5·753
	(2·682)	(3·646)
Tonga (dummy)	–4·961**	–0·331
	(2·268)	(3·459)
Catholic (dummy)	–3·751*	–5·233*
	(2·046)	(2·936)
Instruments		
High quality food products (dummy)	14·463***	16·610***
	(2·370)	(3·571)
Neighbor's shopping mall usage (visits/week)	2·397***	2·146***
	(0·328)	(0·447)
Distance to a shopping mall (km)	–1·132***	–1·622***
	(0·360)	(0·493)
Constant	–75·621***	–88·611***
	(10·594)	(12·721)
Joint significance of instruments: χ^2 (3)	120***	64***
N	930	499

Generalized linear models. Standard errors are reported in parentheses. * $p < 0·10$, ** $p < 0·05$, *** $p < 0·01$.

Table S22. Falsification test for instrument validity (Tobit estimates)

	BMI (kg/m²/Z-score)	HAZ (Z-score)	Calorie (kcal/day)	Protein (g/day)	Iron (mg/day)	Zinc (mg/day)	Vitamin A (µg retinol/day)
	(1)	(2)	(3)	(4)	(5)	(6)	(7)
**Adults**		NA					
High quality food products (dummy)	–0·079		47·512	–0·018	–0·427	–0·073	0·674
	(0·358)		(96·791)	(0·302)	(1·307)	(0·376)	(0·696)
Neighbor's shopping mall usage (visits/week)	–0·004		24·340	0·040	0·250	0·036	–0·057
	(0·051)		(18·738)	(0·053)	(0·205)	(0·058)	(0·090)
Household distance to a shopping mall (km)	0·023		3·231	–0·011	0·032	–0·012	0·012
	(0·052)		(17·223)	(0·049)	(0·240)	(0·040)	(0·065)
Other controls	Yes		Yes	Yes	Yes	Yes	Yes
Joint significance of instruments: F (3,853/919)	0·10		1·02	0·47	1·02	0·36	0·35
Joint significance of instruments: *p*-value	0·958		0·385	0·704	0·382	0·785	0·792
Joint F-statistic	355***		16***	3***	16***	15***	15***
Pseudo-R^2	0·038		0·012	0·044	0·027	0·051	0·070
N	864		930	930	930	930	930
**Children**							
High quality food products (dummy)	–0·275	0·145	–38·936	–0·009	1·068	–2·286	0·456
	(0·441)	(0·529)	(157·455)	(0·302)	(1·508)	(2·022)	(0·692)
Neighbor's shopping mall usage (visits/week)	–0·124**	0·159*	31·934	0·080*	0·126	0·343*	0.036
	(0·062)	(0·083)	(21·533)	(0·045)	(0·143)	(0·191)	(0·076)
Household distance to a shopping mall (km)	–0·080	–0·038	–6.909	–0·025	–0·067	0·091	–0·034
	(0·054)	(0·067)	(15·427)	(0·033)	(0·131)	(0·179)	(0·054)
Other controls	Yes	Yes	Yes	Yes	Yes	Yes	Yes
Joint significance of instruments: F (3, 449/461/488)	1·66	1·23	0·99	1·53	0·46	1·73	0·47
Joint significance of instruments: *p*-value	0·18	0·298	0·398	0·205	0·712	0·159	0·704
Joint F-statistic	355***	··	145***	202***	48***	3***	39***
Pseudo-R^2	0·038	0·165	0·050	0·172	0·139	0·044	0·061
N	460	472	499	499	499	499	499

NA, not applicable. Protein and vitamin A intakes were transformed using an inverse hyperbolic sine transformation: $\ln[\{y + (y^2 + 1)^{0\cdot5}\}]$, in order to retain zero-valued observations. Coefficient estimates are shown with robust standard errors clustered at compound level in parentheses. Other controls: age, age-squared, education, household size, log of income, a dummy (1, 0) variable for male, ethnic groups—Chewa, and Tonga, and religion status—Catholic. * p < 0·10, *** p < 0·01.

References

1. Steyn, N.P.; Nel, J.H.; Nantel, G.; Kennedy, G.; Labadarios, D. Food variety and dietary diversity scores in children: Are they good indicators of dietary adequacy? *Public Health Nutr.* **2006**, *9*, 644–650, doi:10.1079/phn2005912.
2. Wooldridge, J.M. Control function methods in applied econometrics. *J. Hum. Resour.* **2015**, *50*, 420–445, doi:10.3368/jhr.50.2.420.
3. Wooldridge, J.M. *Econometric Analysis of Cross-Section and Panel Data*, 2nd ed; MIT Press: Cambridge, MA, USA, 2010.
4. Umberger, W.J.; He, X.; Minot, N.; Toiba, H. Examining the relationship between the use of supermarkets and over-nutrition in Indonesia. *Am. J. Agric. Econ.* **2015**, *97*, 510–525, doi:10.1093/ajae/aau111.
5. Kimenju, S.C.; Rischke, R.; Klasen, S.; Qaim, M. Do supermarkets contribute to the obesity pandemic in developing countries? *Public Health Nutr.* **2015**, *18*, 3224–3233, doi:10.1017/s1368980015000919.
6. Debela, B.L.; Demmler, K.M.; Klasen, S.; Qaim, M. Supermarket food purchases and child nutrition in Kenya. *Glob. Food Secur.* **2019**, 100341, doi:10.1016/j.gfs.2019.100341.
7. Rischke, R.; Kimenju, S.C.; Klasen, S.; Qaim, M. Supermarkets and food consumption patterns: The case of small towns in Kenya. *Food Policy* **2015**, *52*, 9–21, doi:10.1016/j.foodpol.2015.02.001.
8. Rupa, J.A.; Umberger, W.J.; Zeng, D. Does food market modernisation lead to improved dietary diversity and diet quality for urban Vietnamese households? *Aust. J. Agric. Resour. Econ.* **2019**, *63*, 499–520, doi:10.1111/1467-8489.12308.
9. Di Falco, S.; Veronesi, M.; Yesuf, M. Does adaptation to climate change provide food security? A micro-perspective from Ethiopia. *Am. J. Agric. Econ.* **2011**, *93*, 829–846, doi:10.1093/ajae/aar006.
10. Khonje, M.G.; Qaim, M. Modernization of African food retailing and (un)healthy food consumption. *Sustainability* **2019**, *11*, 4306, doi:10.3390/su11164306.
11. Demmler, K.M.; Ecker, O.; Qaim, M. Supermarket shopping and nutritional outcomes: A panel data analysis for urban Kenya. *World Dev.* **2018**, *102*, 292–303, doi:10.1016/j.worlddev.2017.07.018.
12. Schap, T.; Kuczynski, K.; Hiza, H. Healthy eating index—Beyond the score. *J. Acad. Nutr. Diet.* **2017**, *117*, 519–521, doi:10.1016/j.jand.2017.02.002.
13. Smith, T.A. Do school food programs improve child dietary quality? *Am. J. Agric. Econ.* **2016**, *99*, 339–356, doi:10.1093/ajae/aaw091.
14. Whiteman, E.D.; Chrisinger, B.W.; Hillier, A. Diet quality over the monthly supplemental nutrition assistance program cycle. *Am. J. Prev. Med.* **2018**, *55*, 205–212, doi:10.1016/j.amepre.2018.04.027.
15. FAO. *Human Energy Requirements. Report of a Joint FAO/WHO/UNU Expert Consultation, Food and Nutrition Technical Report No. 1*; Food and Agriculture Organization: Rome, Italy, 2001.
16. WHO, FAO. *Vitamin and Mineral Requirements in Human Nutrition*, 2nd ed.; World Health Organization: Geneva, Switzerland, 2004.
17. Institute of Medicine (IOM). *Dietary Reference Intakes: The Essential Guide to Nutrient Requirements*; The National Academies Press: Washington, DC, USA, 2006.
18. Institute of Medicine (IOM). *Dietary Reference Intakes for Energy, Carbohydrate, Fiber, Fat, Fatty Acids, Cholesterol, Protein, and Amino Acids*; The National Academies Press: Washington, DC, USA, 2002.
19. Brown, K.H.; A Rivera, J.; Bhutta, Z.; Gibson, R.S.; King, J.C.; Lönnerdal, B.; Ruel, M.T.; Sandtröm, B.; Wasantwisut, E.; Hotz, C. International Zinc Nutrition Consultative Group (IZiNCG) technical document #1. Assessment of the risk of zinc deficiency in populations and options for its control. *Food Nutr. Bull.* **2004**, 25, S99–S203.
